# Sliding-window training on readout layer enhances liquid state machines for temporal prediction

**DOI:** 10.1038/s44335-026-00093-1

**Published:** 2026-09-16

**Authors:** Shimeng Wu, Andrew Walter, Andy M. Tyrrell, Martin A. Trefzer

**Affiliations:** School of Physics, Engineering and Technology, University of York, York, UK

**Keywords:** Engineering, Mathematics and computing, Neuroscience

## Abstract

Temporal prediction is essential in scientific and engineering problems, particularly for low-power online inference. Reservoir computing is attractive because a fixed recurrent system maps inputs into a high-dimensional state decoded by a simple readout. Liquid state machines instantiate this idea with recurrent spiking neurons, yielding sparse event-driven states suited to neuromorphic and digital hardware. However, the stepwise protocol trains the readout from an instantaneous state, which can be information-limited when evidence is distributed across time. We improve temporal prediction by providing the readout with a finite temporal context. We concatenate a sliding window of recent reservoir spike states and train on either ridge regression or a transformer readout. On the Nonlinear AutoRegressive Moving Average (NARMA5) benchmark, increasing context length improves both readouts. Median test NRMSE for ridge regression decreases from 2.106 under stepwise decoding to 0.317, an 85% reduction. The transformer readout shows the same trend, decreasing from 0.807 to 0.179, a 78% reduction. The same qualitative trend is further observed on NARMA10 and Mackey Glass prediction tasks. These results demonstrate that exposing recent reservoir evolution at the decoder can substantially reduce the limitations of stepwise decoding while retaining the standard reservoir computing protocol.

## Introduction

Reservoir computing (RC) provides a simple but powerful paradigm for processing temporal data by using a simple trainable readout layer to interpret the behaviour of a fixed dynamical system^1^. Among different forms of RC, the liquid state machine (LSM) uses a biologically inspired spiking neural network (SNN) as the reservoir, instead of the continuous valued units used in classical echo state networks (ESNs)^2,3^. In an LSM, neurons represent and transmit information through discrete and sparse spike events. This event-driven representation aligns naturally with temporal coding in many real-world signals and can improve energy efficiency on neuromorphic or other event-driven hardware by reducing unnecessary activity and data movement for sparse spike workloads^4,5^.

Although recurrent SNNs can now be trained using gradient-based methods such as surrogate gradients^6^, reservoir computing is still attractive due to its simplicity and its practical task adaptation procedure. This makes it useful to distinguish between two ways of using recurrent spiking systems. In one case, the recurrent spiking network itself is treated as the trainable model, so task adaptation requires optimising internal synaptic weights or neuronal parameters. In another case, the recurrent system is treated as a reusable temporal substrate, and task adaptation is performed by changing only the readout.

This is especially relevant for neuromorphic and physical reservoir systems, where the complete internal state of the substrate is not always available. In software simulations, all membrane potentials and synaptic variables can be recorded if needed. By contrast, in deployed neuromorphic hardware, mixed signal substrates, and other physical reservoirs, the most accessible response is often a stream of output spikes or a limited set of externally observable signals, while full neural states, such as membrane potentials and synaptic variables, may be unavailable or difficult to record^7–9^. Surrogate gradient training also typically requires back propagation through the unfolded spiking dynamics, which can introduce substantial memory and computational cost compared with readout training^10^.

In a typical LSM workflow, a continuous input signal is encoded into a time-varying spike train and injected into the reservoir. We then record reservoir activity over time, for example, the output spikes of all neurons, or low-pass filtered spike traces^2,11^, and train a simple readout, typically linear regression or ridge regression, to map reservoir states to task targets. In most implementations, the readout is trained on one state representation per time step, even when that representation is a filtered trace that compresses recent spiking history. This step-by-step training protocol is widely used and easy to implement. However, for sequences with deeper temporal dependencies, interpreting only the reservoir response at a single time step can be insufficient. In that case, the standard stepwise approach implicitly assumes that the instantaneous reservoir state provides a sufficiently informative summary of the recent input history that can be linearly decoded by the readout to predict the current target. Specifically in spiking LSMs, the state is often a sparse spike vector, which is attractive for energy efficiency on event-driven hardware^12,13^, but it can be information-limited at any single step.

Two related issues can contribute to these limitations when the readout is trained only on the instantaneous reservoir state.

1. **A single spike carries almost no information by itself**. In spike-based computation, informative structure often lies in the timing relationships among multiple spikes over short intervals, rather than in one isolated event^14,15^. For time series benchmarks such as Nonlinear AutoRegressive Moving Average (NARMA), it is therefore unrealistic to expect accurate predictions as soon as the reservoir produces its first few spikes in response to the first encoded input sample. Meaningful evidence typically emerges only after several input spikes have been presented and the corresponding reservoir dynamics have developed. This is a general property of reservoir systems, but the limitation is more significant here because the readout observes sparse binary spike states rather than dense real-valued reservoir states.
2. **Information is distributed over time, while the reservoir forgets non-linearly**. Many encoders spread information across a spike train rather than concentrating it into a single moment, so the model must combine recent evidence with short-term history to solve the task. At the same time, a recurrent reservoir exhibits fading memory whose retention depends on parameters, noise, and the geometry of the state space^16–18^. In a spiking reservoir, recurrent interactions and non-linear membrane dynamics mix and distort traces of past spikes. In addition, temporal prediction tasks often require integrating evidence over multiple time steps and exploiting non-linear interactions among past inputs and outputs, even before any spike encoding is applied. If the readout sees only the current state, it must linearly reconstruct task-relevant histories from a representation that already contains a non-linearly decayed mixture of earlier perturbations, while also accounting for the temporal dependencies inherent to the task, which can reduce performance on tasks that require longer or more structured temporal processing.

To address these issues without changing the reservoir itself, we investigate a simple sliding window training method for the readout. Instead of training the readout only on the reservoir state at the current time step, we collect reservoir outputs from the most recent *t*_window_ steps and stack them into a single combined representation. This windowed vector replaces the usual current step reservoir response as the readout input, giving the decoder a wider temporal perspective without introducing any additional physical neurons or changes in the internal reservoir connectivity. Although using a time window increases the readout input dimensionality, the additional computational cost remains manageable. In practice, similar accuracy can often be obtained with a smaller reservoir paired with a longer window.

Conceptually, the past reservoir states can be regarded as virtual reservoirs in time, introducing time as an additional feature dimension while preserving the standard RC separation in which only the readout is trained. The linear windowed readout assigns one weight per reservoir neuron per time step in the window, so even if single spikes are weak carriers of information, the stacked recent states can provide a much richer description of the underlying dynamics. This method also reduces reliance on the reservoir alone to store history through non-linear fading memory, because the readout receives explicit access to several recent reservoir responses.

To explore the temporal structure contained in this reservoir-response window further, we study a transformer-based readout that consumes the same finite context as a short sequence and uses self-attention to aggregate information across time steps^19^. Specifically, at each time step, the transformer receives the most recent *t*_window_ reservoir spike states as a sequence and can attend only within this explicit context window.

**Fig. 1:**
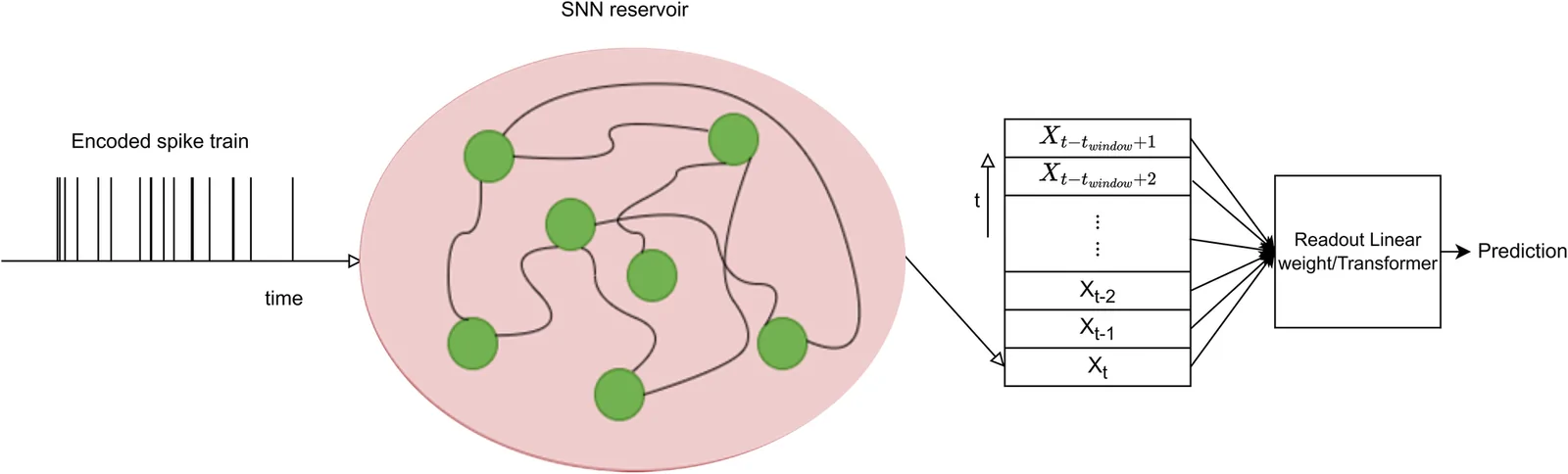
Schematic of the proposed sliding window readout for liquid state machines. An encoded input spike train drives a fixed spiking reservoir. At each simulation step *t*, the readout receives an explicit context of the most recent *t*_window_ reservoir spike state vectors $\{{X}_{t-{t}_{{\rm{window}}}+1},\ldots ,{X}_{t}\}$, which can be consumed either as a concatenated feature vector for ridge regression or as a length *t*_window_ sequence for a transformer readout. In all cases, the reservoir remains unchanged, and only the readout parameters are trained.

Compared to linear regression on a concatenated window, which uses fixed weights across the window, attention can adaptively emphasise the parts of the recent reservoir evolution that are most relevant to the current prediction, while still keeping the reservoir itself fixed. This construction provides an expressive upper-bound probe of the same reservoir states, testing how much structure can be extracted when relations among time steps are modelled flexibly within the explicit context. Figure 1 summarises the proposed sliding-window readout, highlighting that the reservoir dynamics are kept fixed and only the readout consumes an explicit context of the most recent *t*_window_ reservoir states.

From the perspective of temporal structure, our approach is related to classical constructions that introduce explicit temporal context, such as delay coordinate embeddings and tapped delay line style models, which learn from multiple delayed copies of a signal^20,21^. It is also related to delay-based RC implementations that create virtual nodes by time multiplexing a non-linear element with delayed feedback^22–25^. The key difference is that our window operates directly on the high-dimensional reservoir activity rather than on the raw input or a low-dimensional intermediate signal. Instead of attaching an external delay line to compensate for limited memory, we expose a compact view of how the existing recurrent dynamics transform information over a short temporal horizon. In this sense, the windowed reservoir states provide a task-shaped, data-dependent analogue of a tapped delay line, formed after the reservoir’s non-linear transformation.

We investigate whether temporal prediction in liquid state machines (LSMs) can be improved by providing the readout with an explicit, finite temporal context, while keeping the reservoir fixed after an initial unsupervised spike timing dependent plasticity (STDP) stage. This paper make three contribution to the field: (i) we propose a sliding window readout that uses a short history of recent reservoir spike states rather than a single instantaneous state, enabling task specific temporal aggregation at the decoder; (ii) we introduce strictly matched input only baselines that bypass the reservoir but use the same spike encoding, context window definition, and train and test splits, allowing us to separate improvements due to longer explicit history from those due to the reservoir’s nonlinear transformation; and (iii) through this transformer readout analysis, we show that, for the same context length *t*_window_, an attention based readout that processes the window as a sequence exploits this finite context more effectively than fixed linear weighting on the concatenated window, yielding substantially lower error on NARMA5.

## Results

### Sliding-window linear readout on NARMA

**Fig. 2:**
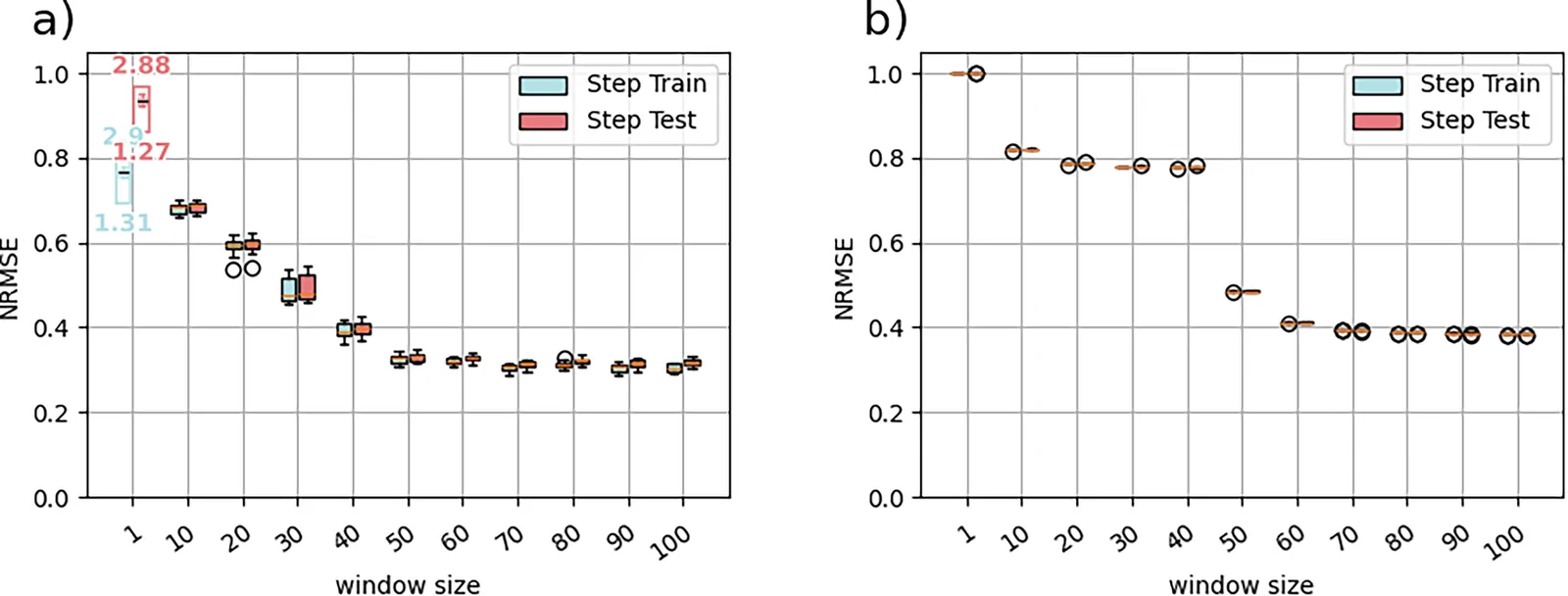
NRMSE (normalized with the standard deviation of the target) on NARMA5 as a function of the temporal context length *t*_window_ (measured in reservoir simulation steps). **a** Sliding window linear readout trained on reservoir spike states, where features are formed by concatenating the most recent *t*_window_ reservoir steps. **b** pure_linear_regression_spike control that bypasses the reservoir and performs linear regression directly on the spike encoded input history with the same *t*_window_ definition. Each box plot aggregates 10 random seeds. To accommodate outliers, the y-axis is displayed with a mixed scale: the main axis shows NRMSE in the range [0, 1], while the few values exceeding 1 are plotted in a compressed top band and annotated with their true NRMSE values (top left of **a**). Both methods improve with a larger *t*_window_, as expected for temporal prediction, but the consistently lower error and the different decay trend of the reservoir-based readout indicate that the reservoir provides a nontrivial computational transformation beyond simply exposing a longer linear history.

Figure 2 reports the NRMSE on Non-linear Auto Regressive Moving Average (NARMA) 5 benchmark under varying temporal context lengths *t*_window_. In all experiments, each NARMA input sample is converted into spikes by a time-to-spike encoding with an encoding window of *W*_enc_ = 10, so one NARMA step corresponds to ten reservoir simulation steps. With the sliding window linear readout trained on reservoir spike states, both training and test errors steadily decrease as *t*_window_ increases. The reduction is approximately linear over *t*_window_ = 10–50, after which the improvement becomes smaller, and the performance approaches a platform. This trend is consistent with the intuition that meaningful information for prediction is distributed across multiple spike events, and that a readout operating on a wider temporal slice of reservoir activity is less constrained by relying on a single instantaneous spike state.

However, because NARMA5 is a temporal prediction benchmark, providing a readout with a longer explicit history can certainly improve the performance. To separate the benefit of simply extending the accessible temporal context for the linear readout from the contribution of the reservoir computation, we introduce a direct linear baseline that bypasses the reservoir. Specifically, we apply the same spike-based encoding to the input sequence, then train a linear regression model directly on a window of the most recent encoded input spikes, using the same context length *t*_window_ and the same train and test data as the reservoir-based experiments. This baseline, therefore, quantifies how far the linear regression itself can go by exploiting longer explicit history alone, without any non-linear mapping from an LSM in the middle.

In this condition, performance also improves as *t*_window_ increases, which confirms that part of the gain in Fig. 2 is a generic consequence of providing a longer history for linear prediction rather than a property unique to LSMs. Nevertheless, the baseline remains consistently worse than the reservoir-based sliding window readout across the entire sweep, and the two curves do not decrease in the same manner. The reservoir-based readout exhibits a near-linear decrease up to *t*_window_ ≈ 50, followed by a gradual saturation, whereas the direct linear baseline barely performs any meaningful prediction before the window size reaches 50 and then quickly reaches its own saturation level. This mismatch indicates that the reservoir is not acting as a passive delay line that merely exposes a longer segment of spikes. Instead, the recurrent spiking dynamics transform the encoded input history into a different representation, one that allows a simple linear readout to extract predictive structure more effectively.

From the perspective of the two issues outlined in the Introduction, the comparison supports both points. First, since a single spike is weakly informative, improvements from increasing *t*_window_ are expected in both conditions because they aggregate more spike evidence. Second, the additional advantage of the reservoir suggests that the non-linear fading memory and recurrent interactions in the liquid reshape this multi-step evidence into a feature space that is better aligned with linear decoding. To complement the aggregate NRMSE, Figs. S8 and S9 in the Supplementary Material provide representative prediction traces and running NRMSE curves for the reservoir readout and the matched input-history baseline.

**Fig. 3:**
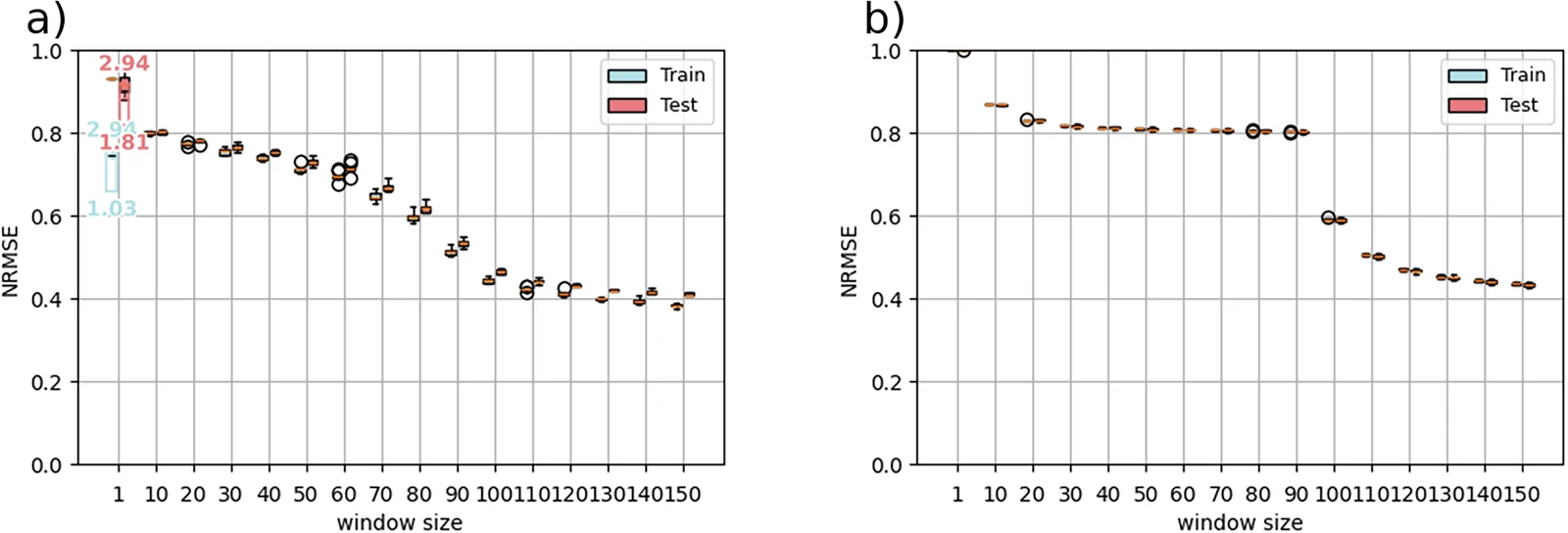
NRMSE on NARMA10 against the temporal context length *t*_window_. **a** The reservoir-based sliding window linear readout. **b** The direct input-history linear baseline using the same spike encoding and window definition. The mixed-scale y axis follows the same convention as Fig. 2. Because NARMA10 has a longer recurrent dependency than NARMA5, the sweep is extended to *t*_window_ = 150 to include the saturation of the performance. The delayed reduction relative to NARMA5 and the clearer separation near the ten-sample scale indicate that the useful temporal context shifts with the memory scale of the task.

We further test the same linear-readout comparison on NARMA10. Since the NARMA10 target is generated from a longer recurrent history than NARMA5, and *W*_enc_ = 10 maps one input sample to ten reservoir simulation steps, we sweep *t*_window_ to 150 so that the readout context covers and exceeds the nominal ten-sample memory scale. The resulting trend is consistent with this longer task memory: the main reduction for the reservoir-based readout occurs later than for NARMA5, and around *t*_window_ = 100 the reservoir readout has already entered a lower-error regime while the direct input-history baseline is still comparatively poor. This supports the same interpretation as in NARMA5, but on a task where the useful context is expected to extend over a longer temporal horizon, as shown in Fig. 3.

### Sliding-window linear readout on Mackey–Glass

**Fig. 4:**
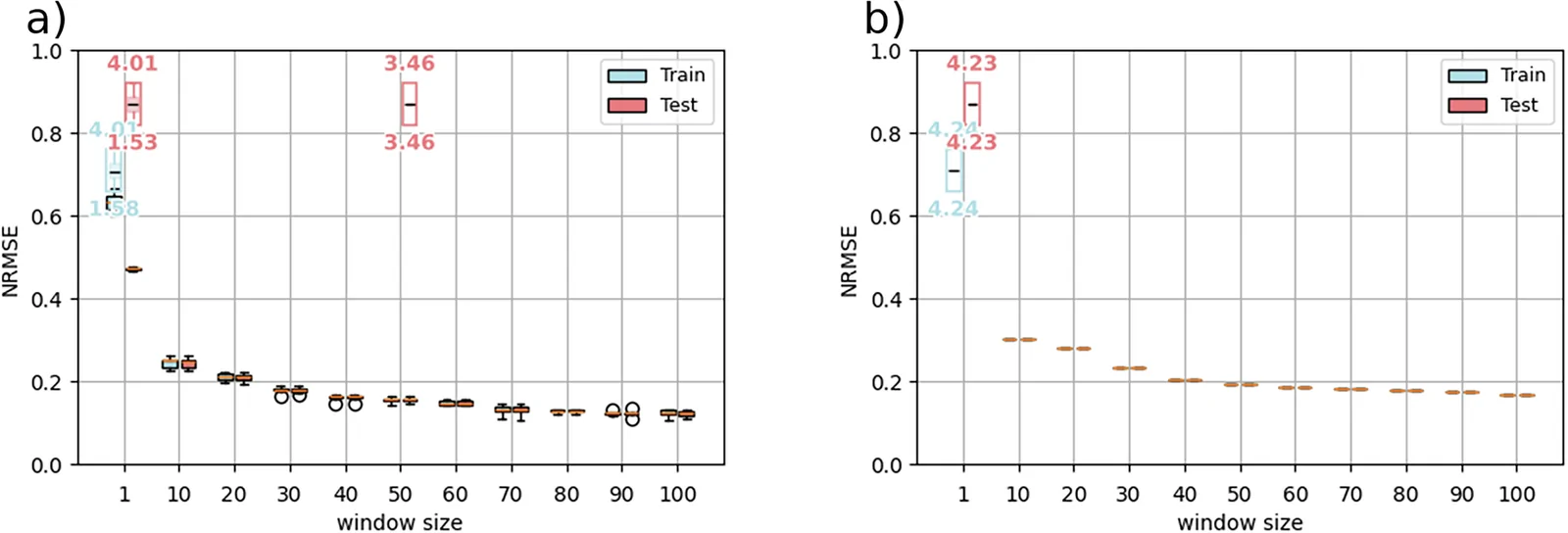
NRMSE for one-step Mackey Glass prediction as a function of temporal context length *t*_window_. **a** The reservoir-based sliding window linear readout. **b** The direct input-history linear baseline using the same spike encoding and window definition. The mixed-scale y axis follows the same convention as Fig. 2. Both methods improve with longer context; the reservoir-based readout improves earlier and is lower through the short and medium window range, while the direct baseline narrows the gap when a long explicit input history is available.

**Fig. 5:**
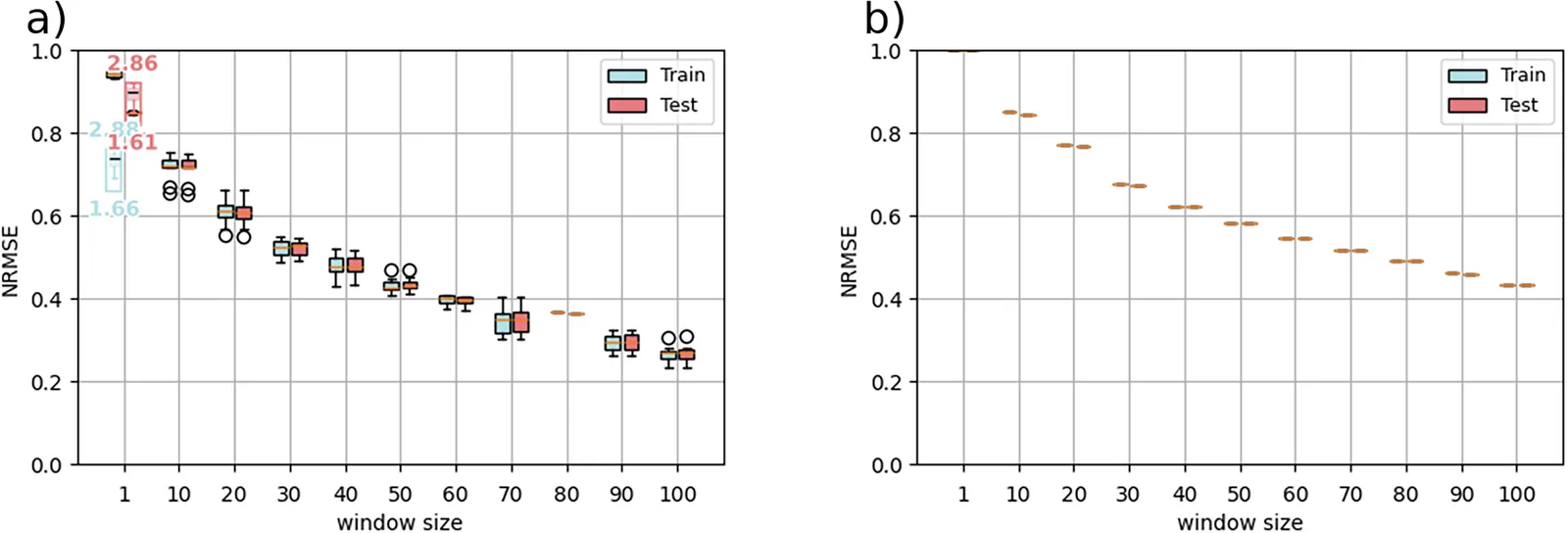
NRMSE for 84-step-ahead Mackey–Glass prediction as a function of temporal context length *t*_window_. **a** The reservoir-based sliding window linear readout. **b** The direct input-history linear baseline using the same spike encoding and window definition. The mixed-scale y-axis follows the same convention as Fig. 2. Compared with the one-step setting, the longer prediction horizon produces a larger and more consistent separation between the reservoir-based readout and the baseline, suggesting that the reservoir transformation is more valuable when the target depends on a longer dynamical history.

We also evaluate Mackey–Glass, a bio-inspired chaotic time-series benchmark^26^, with one-step and 84-step-ahead prediction. In the one-step setting, both the reservoir based readout and the direct input-history baseline improve smoothly as *t*_window_ increases, while the reservoir based readout remains lower across the tested window lengths, as shown in Fig. 4. When the prediction horizon is extended to 84 steps ahead, the separation between the reservoir based readout and the direct input-history baseline becomes larger and more consistent, as shown in Fig. 5.

### Transformer readout on NARMA5

**Fig. 6:**
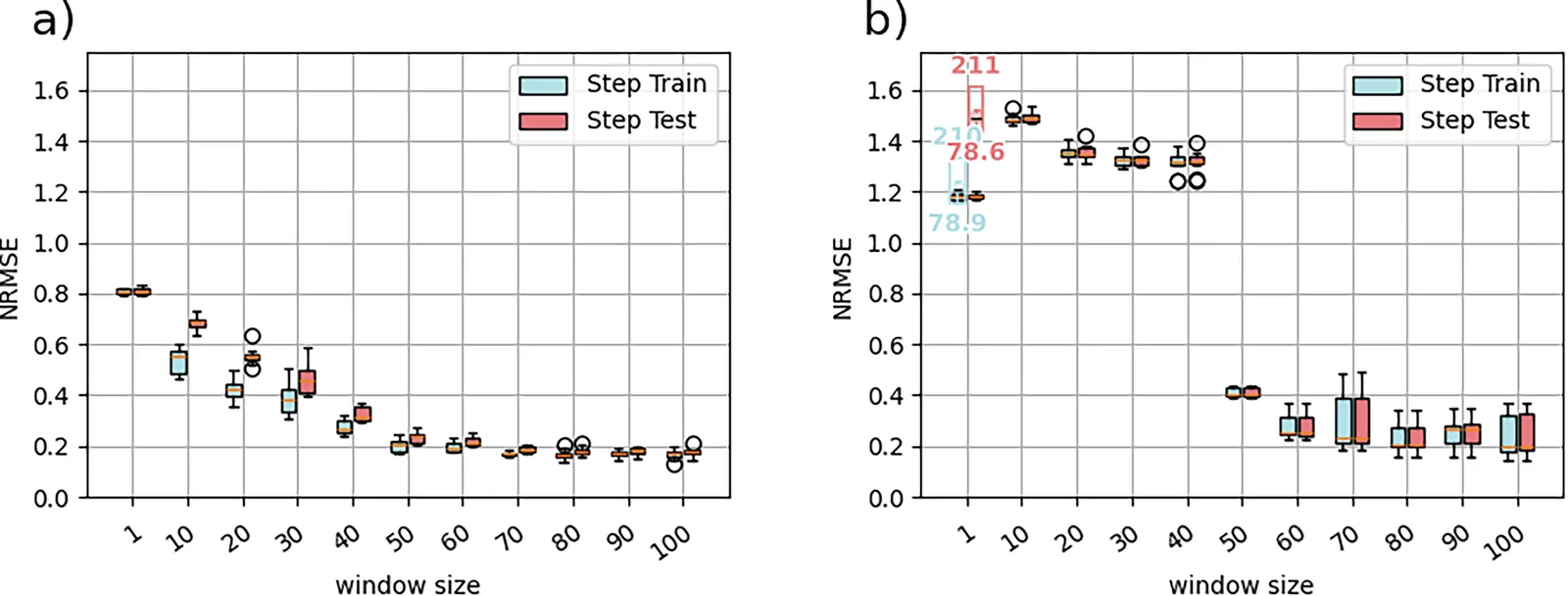
NRMSE versus temporal context length *t*_window_ for two transformer-based conditions. **a** The transformer readout. **b** A pure transformer baseline that bypasses the reservoir and is trained directly on input spike trains. To show both the catastrophic failure at short context and the relative gap once meaningful prediction is reached, the y-axis again uses a mixed scale: the main axis displays NRMSE in [0, 1.75], while values exceeding the range are plotted in a compressed upper band and annotated with their true NRMSE values (top left of **b**).

We next replace the linear readout with a transformer that processes a finite temporal context of reservoir spike states represented as a short sequence. Figure 6 shows that increasing *t*_window_ substantially improves performance at small to medium context lengths. Errors decrease rapidly as the context grows to around *t*_window_ ≈ 50, and then the gains become smaller for larger windows. Compared with the sliding window linear readout, in this upper-bound analysis, the transformer reaches a lower error level and achieves strong performance with a shorter context. This suggests that, beyond simply accumulating more spike evidence, attention helps the readout focus on the most informative parts of the recent reservoir evolution instead of relying on a fixed weighting across time. The Supplementary Material also includes a matched MLP readout control (Fig. S3) to separate the effect of a generic nonlinear readout from the temporal weighting provided by self-attention.

Similarly, to isolate the effect of exposing longer explicit history from the effect of reservoir computation, we evaluate a pure transformer baseline that bypasses the reservoir and uses the same spike encoded input history, the same *t*_window_ definition, and the same train and test splits. The baseline performs a random guess only on short contexts. Meaningful prediction emerges only once the context covers the scale of five NARMA steps. Even in this region, the baseline remains consistently worse and more variable than the reservoir with transformer readout. The gap indicates that the reservoir is not merely exposing a longer input history. Instead, its recurrent spiking dynamics transform the encoded input into a representation that is easier to decode, while the transformer further benefits from explicitly aggregating information across recent reservoir states.

## Discussion

The results show that exposing a short temporal context at the readout can substantially improve temporal prediction in liquid state machines, even when the way the reservoir is generated is unchanged. When *t*_window_ = 1, the readout reduces to the standard stepwise approach that decodes from a single per-step reservoir representation. In this situation, performance is limited, which is consistent with the view that informative evidence in spike-based computation accumulates over multiple events and that the current reservoir state provides only a compressed and non-linear mixture of recent history. Increasing *t*_window_ makes more of the reservoir evolution directly available to the readout layer and reduces the requirement of reconstructing history from a single instantaneous snapshot. The window length at which the improvement begins to saturate is therefore expected to depend on the temporal scale of the benchmark. For NARMA tasks, this scale is related to the recurrence order after spike encoding: with *W*_enc_ = 10, the change in behaviour around *t*_window_ ≈ 50 corresponds to roughly five NARMA steps and is consistent with the effective order of NARMA5, while the longer recurrence order of NARMA10 is consistent with the improvement continuing over a larger part of the sweep.

A key observation is that the improvement from a larger *t*_window_ is not explained by longer explicit history alone. The pure baselines, which apply the same window definition to the spike encoded input while bypassing the reservoir, also benefit from increasing context, confirming that some gain is generic. However, across the sweep, the reservoir-based readouts remain consistently better and exhibit a different trend. This persistent gap indicates that the liquid is not acting as a passive delay line that merely exposes past inputs. Instead, the recurrent spiking dynamics reshape the encoded input history into a representation that is easier to decode. A corresponding input-side tapped-delay-line control is reported in Fig. S10 of the Supplementary Material, and it does not reproduce the gain obtained by exposing recent reservoir states directly to the readout.

The comparison between the linear windowed readout and the transformer readout further clarifies what is gained by different ways of using the same finite context. In this comparison, the transformer acts as an expressive upper-bound probe of the same reservoir-response window, allowing the analysis to test how much task-relevant temporal structure remains extractable when relations among states in the window are modelled flexibly. Linear regression on the concatenated window assigns one fixed weight per neuron per time step. This is sufficient to extract useful structure once enough temporal evidence is available, and the smooth improvement with context suggests that much of the relevant information is linearly decodable when the readout is given an explicit view of recent reservoir states. The transformer achieves a lower error floor and reaches strong performance with shorter contexts, which suggests that the mapping from recent reservoir dynamics to the target benefits from time-dependent weighting. Attention can emphasise the most informative parts of the recent reservoir response and downweight less useful steps, which is difficult for a fixed linear weighting.

These findings also help position the method relative to common spike-based state representations such as low-pass filtered traces. Filtering provides a fixed form of short-term integration by compressing recent spiking history into a continuous value per neuron. This can improve stability compared with raw binary states. However, it is also a lossy compression that mixes temporal structure according to a predetermined kernel. By contrast, the sliding window preserves step-resolved information within the chosen horizon and allows the readout to learn a task-specific temporal weighting over that scope. The linear window can be seen as learning an optimal temporal filter for each neuron under a ridge-regularised objective, while the transformer generalises this to content-dependent and context-dependent weighting. In tasks where fine temporal structure matters, preserving this resolution can be advantageous compared with relying solely on a fixed low-pass kernel.

From a deployment perspective, the proposed sliding window linear regression has an attractive property when the reservoir state is represented as binary spikes. Inference for a linear readout reduces to accumulating weights associated with active features rather than performing general multiplications, since each feature is 0 or 1. This can be implemented as conditional additions and weight lookups, and it naturally benefits from spike sparsity because computation can be event-driven. The main cost shifts to storing the windowed state and the expanded readout weights. In practice, this trade-off can be favourable on digital hardware where multipliers are significantly more expensive than memory.

From a systems perspective, this introduces a practical accuracy against a compute trade-off. Training of both the linear regression and transformer readouts is naturally parallelisable and can benefit from GPU acceleration. In contrast, reservoir simulation and reservoir response collection are inherently streaming, due to spiking neuron dynamics and recurrent connectivity. As reservoir size increases, throughput can degrade sharply because of recurrent event propagation, event handling overhead, and increased memory traffic. This asymmetry suggests that, when comparable accuracy can be achieved with a smaller reservoir paired with a longer context window, shifting more computation effort to the readout temporal context, rather than scaling up the recurrent reservoir, can be an effective route to improve efficiency. Figure S11 of the Supplementary Material further compares reservoir size against readout context, showing that simply enlarging the recurrent reservoir with a shorter readout context is less effective than using a compact reservoir with a longer readout window in this setting.

To place the proposed method in context, Table 1 summarizes a set of representative results reported for recurrent-network and reservoir-computing baselines on related temporal prediction tasks. Fully trainable ERNN/LSTM/GRU models can achieve lower errors^27^, but they train recurrent parameters rather than keeping the reservoir fixed. Within the readout-only setting, our sliding-window LSM lies between a standard ESN baseline and a weaker NARX/tapped-delay recurrent baseline on NARMA10, and is lower than the small LSTM baseline reported for 84-step Mackey–Glass. The CMOS SNN reservoir result is included because it is close to our hardware-oriented setting, but its reported NARMA10 value uses a mean-normalized NRMSE, which is typically smaller than target variance normalized, and therefore should be treated as contextual rather than directly comparable.

**Table 1:** Representative comparison of reported NRMSE values on related temporal-prediction benchmarks

Method/system	Class	NRMSE norm.	NARMA 5	NARMA 10	MG 1-step/short	MG 84-step
This work: LSM + sliding-window ridge readout	LSM	RMSE/*σ*_*y*_	**0.317**	**0.410**	**0.124**	**0.272**
ERNN/Elman network^27^	Trainable RNN	RMSE/*σ*_*y*_	n.r.	0.075	0.060^a^	n.r.
LSTM^27,37^	Trainable gated RNN	RMSE/*σ*_*y*_	n.r.	0.072	0.033^a^	0.470
GRU^27^	Trainable gated RNN	RMSE/*σ*_*y*_	n.r.	0.092	0.033^a^	n.r.
NARX^27^	Tapped-delay recurrent model	RMSE/*σ*_*y*_	n.r.	0.536	0.073^a^	n.r.
Standard ESN^38^	ESN	RMSE/*σ*_*y*_	n.r.	0.245 ± 0.020	n.r.	0.201 ± 0.029
Connected hierarchical ESN^39^	Hierarchical ESN	RMSE/*σ*_*y*_	0.22	0.36	n.r.	n.r.
Unconnected hierarchical ESN^39^	Hierarchical ESN	RMSE/*σ*_*y*_	0.30	0.40	n.r.	n.r.
CMOS field-programmable SNN reservoir^40^	Hardware SNN reservoir	RMSE/$\bar{y}$	n.r.	≈0.20	n.r.	n.r.

Values are not a strict ranking because input range, prediction horizon, train/test split, model size, and optimization protocol differ across sources. “n.r.” indicates not reported. The CMOS SNN reservoir row reports RMSE/$\bar{y}$, where $\bar{y}$ is the mean teaching signal over the test interval, rather than RMSE/*σ*_*y*_; for positive NARMA targets, this convention uses a much larger denominator than our normalization and can make the reported NRMSE numerically much smaller, so that row should not be directly compared against ours.

^a^Bianchi et al.^27^ use Mackey–Glass with a forecast horizon *t*_*f*_ = 12, so these values are short-horizon references rather than exact one-step or 84-step comparisons.

Overall, the results support a simple interpretation. Many of the limitations of stepwise decoding in spiking reservoirs arise because informative evidence is distributed across multiple simulation steps and is non-linearly mixed in the current state. Providing the readout with an explicit view of reservoir response improves performance without changing the reservoir, and attention-based decoders can further exploit the same finite context by learning which parts of the recent response matter most for the prediction.

These results motivate several directions for future work. First, we will evaluate sliding window and transformer readouts on a broader range of temporal prediction tasks, such as the Santa Fe laser dataset, Japanese vowels dataset^26^, and on more realistic event-driven use cases such as program deviation detection^28^. Second, we will study how the choice of spike encoding shapes the effective temporal context available to the readout and the sparsity of reservoir activity^29^. Third, we will target hardware deployment of the windowed linear readout on digital platforms, exploring the performance consistency between software inference and fixed-point implementation, and quantifying the resource cost introduced by the reservoir and the readout layer. The present experiments use conventional ridge regression to keep the linear readout simple; for hardware-oriented deployment, future work will also test *L*_1_ regularization of the same windowed readout to prune unused weights and reduce storage and additions at inference.

## Methods

This work follows the standard reservoir computing separation. A scalar input *u*[*n*] is first converted into spikes and injected into a fixed spiking reservoir. We record the reservoir spike activity over time and train only the readout to predict the target *y*[*n*]. We evaluate two readout designs, a sliding window linear readout and a transformer readout, and we include matched pure baselines that bypass the reservoir and operate directly on the spike encoded input history under the same context definition.

### Reservoir generation and simulation

We evaluate the readout on NARMA and Mackey–Glass temporal prediction benchmarks. For NARMA, a scalar input sequence *u*[*n*] is generated following the standard NARMA protocol, and the objective is to predict the corresponding target sequence *y*[*n*] at each discrete step *n*. In particular, we use NARMA5 and NARMA10 systems in the standard form:

1 $$y[n+1]=0.3\,y[n]+0.05\,y[n]\left(\mathop{\sum }\limits_{i=0}^{K-1}y[n-i]\right)+1.5\,u[n-K+1]\,u[n]+0.1,$$

where *K* = 5 for NARMA5 and *K* = 10 for NARMA10. The input *u*[*n*] is sampled uniformly from [0, 0.5], and *y*[*n*] = 0 for *n* < 0 as initial conditions^25^.

We also use the Mackey–Glass system as a bio-inspired chaotic time-series benchmark^26^. The continuous signal is generated from the delay differential equation

2 $$\frac{dx(t)}{dt}=\beta \frac{x(t-\tau )}{1+x{(t-\tau )}^{q}}-\gamma x(t),$$

where *β* = 0.2, *γ* = 0.1, *q* = 10, and *τ* = 17 are the standard Mackey–Glass parameters. After numerical integration, the sampled scalar sequence *x*[*n*] is used as the input signal, and the readout is trained to predict *x*[*n* + *h*], with *h* = 1 for one-step prediction and *h* = 84 for the longer horizon experiment.

**Fig. 7:**
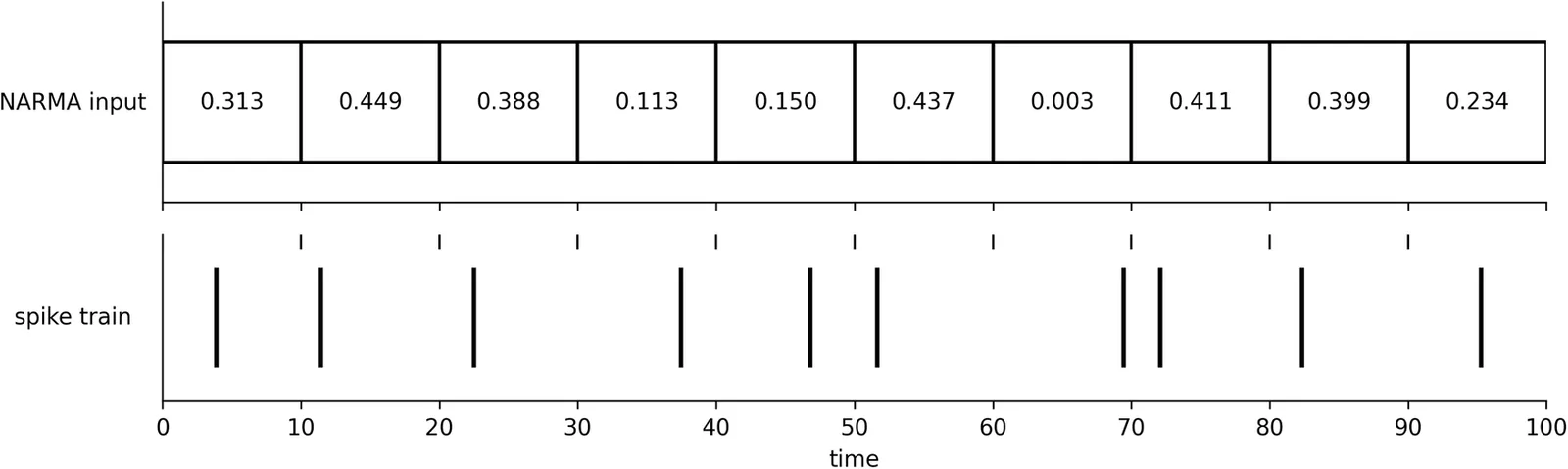
Illustration of the time to spike encoding used in this work. Each discrete NARMA input sample *u*[*n*] is kept constant over an encoding window of length *W*_enc_ simulation steps, and is mapped to exactly one spike within that window. The spike time is determined by the magnitude of *u*[*n*], the larger the value is, the earlier the spike will be put in the window, vice versa.

To match the event-driven operation of the liquid state machine (LSM), the continuous valued input *u*[*n*] is converted into a short spike pattern by a time to spike encoding^29^. Throughout this work, we use an encoding window of *W*_enc_ = 10, such that each NARMA step corresponds to ten simulation steps of the spiking reservoir. For each *u*[*n*], we generate a single spike whose timing is placed within {1,…, *W*_enc_} according to the encoded magnitude, as illustrated in Fig. 7. And this encoded spike train is broadcast to all reservoir neurons via input synapses with randomly generated input weights. Figures S4 and S5 in the Supplementary Material test whether the choice of *W*_enc_ = 10 creates a significant resolution bottleneck, while Figs. S6 and S7 repeat the sliding-window readout experiment with rate and thermometer encodings.

The spiking reservoir is simulated in Brian2^30^ using a leaky integrate and fire (LIF) model:

3 $$\frac{{\rm{d}}{v}_{i}(t)}{{\rm{d}}t}=\frac{{v}_{{\rm{rest}}}-{v}_{i}(t)}{{\tau }_{{\rm{leak}}}},$$

4 $${v}_{i}({t}^{+})=\left\{\begin{array}{ll}{v}_{i}({t}^{-})+{w}_{ij},\quad &{\rm{if\,neuron}}\,j\,{\rm{spikes\,at\,time}}\,t,\\ {v}_{i}({t}^{-}),\quad &{\rm{otherwise}}.\end{array}\right.$$

with *v*_rest_ = 0, *τ*_leak_ = 6 ms, threshold *v*_th_ = 1, and no refractory period. After firing a spike, the membrane potential is reset to *v*_rest_. The reservoir size is fixed to 400 neurons in all reported results. Figure S1 of the Supplementary Material reports a leak-constant sweep to test whether adjusting the reservoir timescale alone could account for the gain from the readout-side temporal window.

Reservoir connectivity follows an exponential spatial topology, bio-inspired and consistent with the exponential distance rule (EDR)^31–33^. Each neuron *i* is assigned a position (*x*_*i*_, *y*_*i*_) on a 2D plane. Given two neurons *i* and *j*, their distance is computed as

5 $${d}_{ij}=\sqrt{{({x}_{i}-{x}_{j})}^{2}+{({y}_{i}-{y}_{j})}^{2}},$$

For *i* ≠ *j*, a directed synapse *i* → *j* is created independently with probability

6 $$P(i\to j)=\exp \left(-\frac{{d}_{ij}}{s}\right),$$

where *s* = 0.03. 50% of the synapse weights are randomly initialized as excitatory synapses between 0 and 1.5 and 50% are inhibitory between -1.5 and 0.

Each run uses a total of *N*_total_ = 350,000 NARMA steps, with *N*_wash_ = 35,000 steps for washout, *N*_train_ = 265,000 steps for training, and the remaining *N*_test_ = 50,000 NARMA steps for testing. Because *W*_enc_ = 10, the corresponding numbers of simulation steps are *T*_total_ = 3,500,000, *T*_wash_ = 350,000, *T*_train_ = 2,650,000, and *T*_test_ = 500,000. The Brian2 simulation step *dt* is set to 1 ms.

Spike timing dependent plasticity (STDP) is enabled during the early stage of the washout period to optimise the connectivity of the reservoir, and is then frozen for readout training and testing. Here, plasticity is applied only to the recurrent synapses, while the input projection remains fixed. This brief, input-driven adaptation stage can be viewed as a lightweight self-organisation step that shapes the liquid dynamics before we record states for readout learning. We use a standard pair-based STDP rule implemented with exponentially decaying pre- and post-eligibility traces^34,35^. For each neuron *i* we maintain a pre trace ${a}_{i}^{{\rm{pre}}}(t)$, and for each neuron *j* we maintain a post trace ${a}_{j}^{{\rm{post}}}(t)$, with dynamics:

7 $$\frac{{\rm{d}}{a}_{i}^{{\rm{pre}}}(t)}{{\rm{d}}t}=-\frac{{a}_{i}^{{\rm{pre}}}(t)}{{\tau }_{{\rm{pre}}}},$$

8 $$\frac{{\rm{d}}{a}_{j}^{{\rm{post}}}(t)}{{\rm{d}}t}=-\frac{{a}_{j}^{{\rm{post}}}(t)}{{\tau }_{{\rm{post}}}}.$$

Where ${\tau }_{{\rm{pre}}}={\tau }_{{\rm{post}}}=20\,{\rm{ms}}$. When a presynaptic spike from neuron *i* arrives at a synapse *i* → *j*, the pre trace is increased by a constant ${A}_{{\rm{pre}}}=0.01$ and the synaptic weight is updated in proportion to the current post trace,

9 $${a}_{i}^{{\rm{pre}}}\leftarrow {a}_{i}^{{\rm{pre}}}+{A}_{{\rm{pre}}},$$

10 $${w}_{ij}\leftarrow {w}_{ij}+{a}_{j}^{{\rm{post}}}.$$

When neuron *j* emits a postsynaptic spike, the post trace is increased by a constant *A*_post_ = − 0.0105 and the synaptic weight is updated in proportion to the current pre trace,

11 $${a}_{j}^{{\rm{post}}}\leftarrow {a}_{j}^{{\rm{post}}}+{A}_{{\rm{post}}},$$

12 $${w}_{ij}\leftarrow {w}_{ij}+{a}_{i}^{{\rm{pre}}}.$$

We record the reservoir activity as a binary spike state matrix $X\in {\{0,1\}}^{{T}_{{\rm{total}}}\times N}$, where *X*_*t*,*i*_ = 1 if neuron *i* spikes at simulation step *t*, and 0 otherwise. This binary spike state is used directly as the readout input.

### Readout methods

Both readouts use the same temporal context definition. For a given *t*_window_, the readout receives the most recent *t*_window_ spike state vectors $\{{X}_{t-{t}_{{\rm{window}}}+1,:},\ldots ,{X}_{t,:}\}$.

#### Sliding window linear readout

We form an extended state *S*_*t*_ by concatenating the most recent *t*_window_ spike state vectors:

13 $${S}_{t}=[{X}_{t-{t}_{{\rm{window}}}+1,:}\,\ldots \,{X}_{t,:}]\in {\{0,1\}}^{1\times (N\cdot {t}_{{\rm{window}}})}.$$

Stacking these over the training interval yields $M\in {{\mathbb{R}}}^{{T}_{{\rm{train}}}\times (N\cdot {t}_{{\rm{window}}})}$. Let *Y*_train_ be the corresponding target vector aligned to the same simulation steps. We solve ridge regression:

14 $$w={({M}^{\top }M+\lambda I)}^{-1}{M}^{\top }{Y}_{{\rm{train}}},$$

with a fixed regularisation coefficient *λ* = **1**. A ridge-regularisation sweep in Fig. S2 of the Supplementary Material verifies that the main trends are not driven by the fixed choice of *λ* = 1.

#### Transformer readout

We use a transformer encoder as the readout that attends over the most recent *t*_window_ reservoir spike states. The reservoir remains fixed, and only the readout parameters are trained. The transformer has 1 encoder layer, 4 attention heads, model dimension 128, feedforward dimension 512, dropout 0.1, learning rate 10^−3^, and trained for 300 epochs for each seed. The transformer readout is implemented using the PyTorch transformer module^36^.

## Supplementary information

Supplementary Information

## Data Availability

No external datasets were used in this study. All data supporting the findings were generated algorithmically from the NARMA and Mackey–Glass process described in Eqs (1) and (2), and the experimental protocol described in Methods.

## Code availability

The code used to generate the results reported in this study is not publicly available, but may be provided to researchers for the purpose of reproducing the published results upon reasonable request from the corresponding author.
