# Supplementary material for: Sliding-window training on readout layer enhances liquid state machines for temporal prediction
Source: Npj Unconv Comput. 2026 Sep 16;3(1):52. doi: 10.1038/s44335-026-00093-1 (PMC13581606; doi:10.1038/s44335-026-00093-1)
Supplement: Supplementary file 1 [file 44335_2026_93_MOESM1_ESM.pdf]

Supplementary Material for: Sliding-Window Training on readout layer Enhances Liquid State Machines for Temporal Prediction

Shimeng Wu[1*], Andrew Walter[1], Andy M. Tyrrell[1], Martin A. Trefzer[1]

[1]School of Physics, Engineering and Technology, University of York, York, UK.

*Corresponding author(s). E-mail(s): shimeng.wu@york.ac.uk;

Tau sweep

The main manuscript uses a fixed leaky integrate and fire membrane leak time constant of $\tau_{\text{leak}} = 6$ ms. To test whether the performance improvement from the sliding window readout could instead be achieved simply by tuning this intrinsic reservoir timescale, we swept τ_{leak} while evaluating two temporal context settings: $t_{\text{window}} = 1$ and $t_{\text{window}} = 50$.

The case $t_{\text{window}} = 1$ corresponds to the stepwise readout setting, where the readout receives only the current reservoir spike state. The case $t_{\text{window}} = 50$ corresponds to a finite temporal context covering five NARMA input steps under the encoding window $W_{\text{enc}} = 10$.

Figure S1 shows that tuning τ_{leak} alone is not sufficient to overcome the limitation of the stepwise readout. When $t_{\text{window}} = 1$, the model fails to produce accurate predictions over the tested leak constants. This suggests that the poor performance of the stepwise readout is not simply due to a poorly chosen membrane leak time constant.

When $t_{\text{window}} = 50$, the performance is much better across a wide range of leak constants. Fine tuning τ_{leak} can further improve the absolute NRMSE, but this improvement is secondary compared with the gain obtained by increasing the readout context from one reservoir state to a finite window of reservoir states. In other words, leak time constant tuning changes the reservoir operating point, while the sliding window readout addresses a different limitation: the lack of explicit temporal context at the decoder.

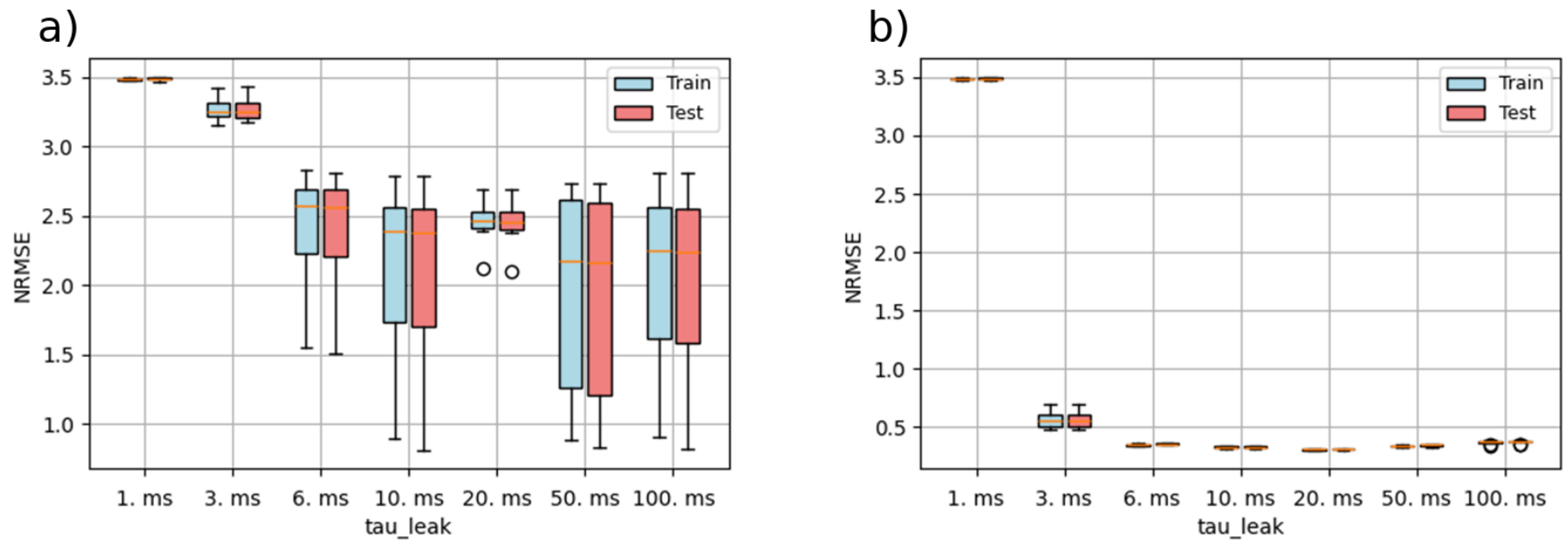

Figure S1: Sensitivity of NARMA5 prediction performance to the LIF leak time constant τ_{leak}. a) Sliding window ridge readout with $t_{\text{window}} = 1$. b) Sliding window ridge readout with $t_{\text{window}} = 50$. Each box summarizes 10 random seeds. For $t_{\text{window}} = 1$, changing τ_{leak} does not lead to effective prediction, and both training and test NRMSE remain high across the tested range. For $t_{\text{window}} = 50$, the absolute performance depends on τ_{leak}, but the readout achieves substantially lower NRMSE than the stepwise setting across a broad range of leak constants.

The value $\tau_{\text{leak}} = 6$ ms used in the main manuscript is therefore a reasonable representative setting rather than a uniquely optimized value. The main conclusion does not depend on the stepwise model being evaluated at an selected poor leak time constant.

Lambda sweep

The main manuscript uses ridge regression with a fixed regularisation coefficient $\lambda = 1$. Since the dimensionality of the sliding-window readout increases with t_{window}, we evaluated whether the reported performance depends strongly on this fixed choice of regularisation strength.

We swept the ridge regularisation coefficient at three representative window sizes, $t_{\text{window}} = 1$, 50, and 100, corresponding to small, intermediate, and large readout feature dimensions. For $t_{\text{window}} = 1$ and $t_{\text{window}} = 100$, we tested $\lambda \in \{10^{-3}, 10^{-2}, 10^{-1}, 1, 10, 100\}$. For $t_{\text{window}} = 50$, values below $\lambda = 0.1$ were also tested, starting from 10^{-3}, but these weak-regularisation settings produced NaN values during training across the 10 seeds and are therefore omitted from the figure.

Figure S2 shows that the main conclusions are not sensitive to the exact value of λ. At $t_{\text{window}} = 1$, changing λ does not recover the low NRMSE obtained with longer temporal windows, suggesting that the poor performance is mainly caused by insufficient temporal context rather than excessive regularisation. At $t_{\text{window}} = 50$ and $t_{\text{window}} = 100$, different λ values produces only small differences in both training and test NRMSE, and the default value $\lambda = 1$ performs comparably to the other tested values.

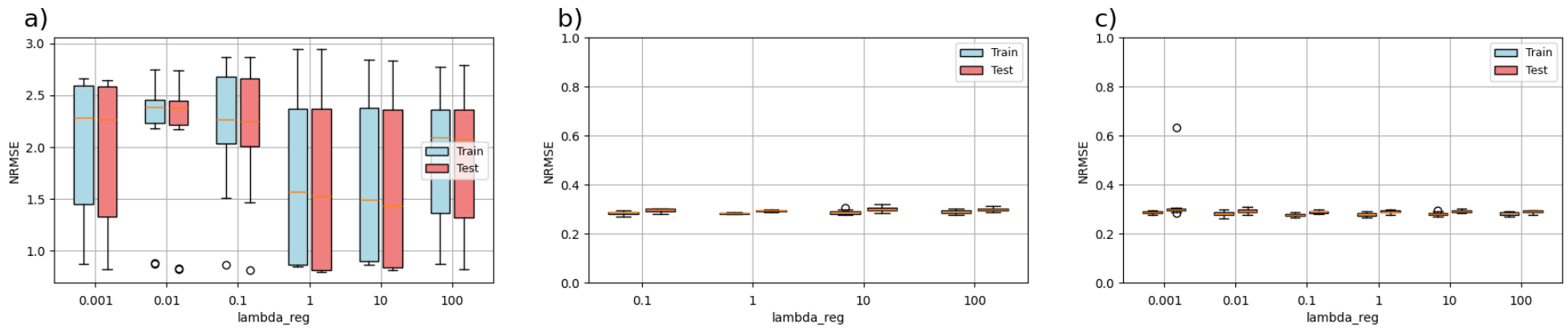

Figure S2: Sensitivity of NARMA5 prediction performance to the ridge regularisation coefficient λ at three representative sliding-window sizes. a) $t_{\text{window}} = 1$. b) $t_{\text{window}} = 50$. c) $t_{\text{window}} = 100$. Each box summarizes 10 random seeds. At $t_{\text{window}} = 1$, the NRMSE remains high across the tested values of λ, indicating that the poor performance of the single-state readout is mainly due to insufficient temporal context rather than the underfitting caused by high regularisation penalty. At $t_{\text{window}} = 50$ and $t_{\text{window}} = 100$, the train and test NRMSE remain similar across the range of λ, and the default value $\lambda = 1$ used in the paper performs comparably to the other tested values. Values below $\lambda = 0.1$ for $t_{\text{window}} = 50$ produced NaN values during training across the 10 seeds and are therefore omitted in the plot. Y-axis limits differ between panels for visual clarity.

Overall, the sweep indicates that the reported improvement is not driven by careful tuning of λ, but by the use of a finite temporal context in the readout.

MLP readout vs Transformer readout

To test whether the improvement of the transformer readout can be explained simply by using a nonlinear readout, we performed an additional ablation using a multilayer perceptron (MLP) readout. The MLP was evaluated on NARMA5 with the same reservoir spike states, the same train and test split, and the same context length of $t_{\text{window}} = 50$ as the transformer comparison. The experiment was repeated over 10 random seeds. The MLP used the same basic depth, hidden layer dimension, and activation function (GELU) as the transformer readout.

This ablation is motivated by the possibility that the lower error of the transformer readout, compared with the linear windowed readout, may come from non-linear transformations of the reservoir states rather than from attention alone. This is related to earlier ESN observations that non-linear readout expansions, such as squared reservoir states, can improve performance [1]. In the present work, however, the raw reservoir states are binary spike states, so element wise squaring would be degenerate because $x^2 = x$ for $x \in \{0, 1\}$. The MLP therefore provides a simple non-linear readout control on the same reservoir state window.

Figure S3 shows that the MLP readout does not reproduce the transformer's performance. The median error of the MLP remain much higher than those of the transformer, and in our runs the MLP also performs worse than the sliding window ridge readout reported in the main manuscript at the same context length. This shows that more complex model does not necessarily improve prediction in this setting. The

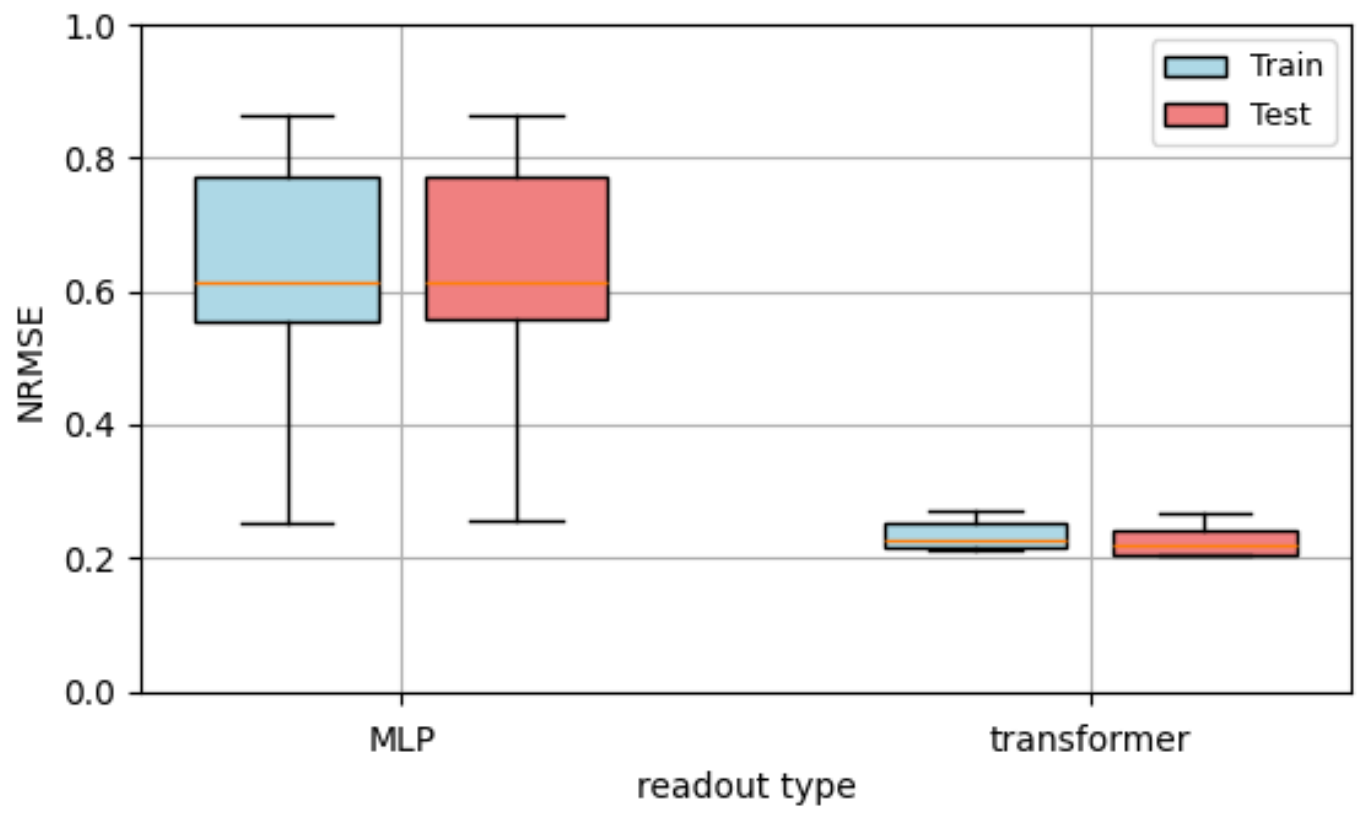

Figure S3: Comparison between an MLP readout and a transformer readout on NARMA5 with $t_{\text{window}} = 50$. Both readouts use the same reservoir spike state window and the same train and test split. Each box summarises 10 random seeds. The MLP shows substantially higher median train and test NRMSE than the transformer, although the best MLP runs approach the transformer range. This suggests that a feed forward non-linear readout can occasionally extract useful structure from the reservoir state window, but with much less reliability than the transformer readout.

windowed reservoir representation is a high dimensional vector: for $N = 400$ neurons and $t_{\text{window}} = 50$, the flattened MLP input has 20,000 binary features. The sliding window linear readout has no learned hidden bottleneck: each neuron time feature can contribute directly to the output through its fitted coefficient, while ridge regularisation controls the size and stability of these coefficients. The MLP, by contrast, must first compress the full sparse window into a hidden layer representation before the output can be predicted.

Therefore, a likely contributing factor is the mismatch between the flattened input dimensionality and the width of the MLP hidden layer. With $t_{\text{window}} = 50$, the first MLP layer maps 20,000 binary neuron time features into only 128 hidden layer nodes. It may cause a strong representation bottleneck, as the network must learn which neurons, temporal positions, and cross time combinations should be preserved in a 128 dimensional summary of the whole window. If this compression does not preserve the task relevant sparse features, later non-linear layers cannot recover them. This can make a compact MLP worse than the linear readout, even though the MLP is theoretically more expressive.

Encoding resolution controls

The main experiments use a time to spike encoding window of $W_{\mathrm{enc}} = 10$. With this encoding, each scalar NARMA input sample is represented by one spike, and the input value is expressed by the spike timing inside the encoding window. This introduces a finite timing resolution. We therefore tested whether this finite resolution, by itself, makes the NARMA5 prediction problem substantially harder in the present setting.

We performed two additional control experiments. First, we increased the time to spike encoding window in the reservoir based sliding window linear readout. For each setting, the readout window was scaled with the encoding window, so that the readout covered the same number of original NARMA input samples. This tests whether a finer time to spike representation improves the complete reservoir based model when the reservoir parameters are kept fixed. However, changing W_{enc} is not only a change in input resolution. It also changes the temporal pattern of spikes injected into the fixed LIF reservoir. Therefore, a different W_{enc} may require re-tuning parameters such as τ_{leak}, v_{th}, or the input scaling. For this reason, we also performed a second control that removes the reservoir and directly compares spike encoded and numerical input histories under the same input only linear readout.

In the second control, we removed the spike encoding from the pure linear regression baseline and trained the same input only linear model directly on the original numerical NARMA input history. This numerical input control removes the finite spike timing resolution introduced by the time to spike encoder, while also removing any coupling between the encoder and the reservoir dynamics. Therefore, if this finite resolution made NARMA5 much harder to predict, the numerical input control should be clearly better than the spike based input control.

Figure S4 shows the reservoir based result. Increasing W_{enc} from 10 to 20 or 30 does not improve the prediction performance. The larger encoding windows give similar or slightly worse NRMSE. This shows that, with the reservoir parameters used in the main experiments, increasing the number of available spike timing positions is not sufficient to reduce the error. The result therefore does not support the interpretation that the $W_{\mathrm{enc}} = 10$ timing resolution is the main bottleneck in the current reservoir based setup.

Figure S5 shows the input only linear regression control. Removing the spike encoding and using the original numerical input values gives only a small change in NRMSE. The numerical input baseline is slightly better, which is expected because it has direct access to the continuous input values. However, the important point is that this improvement is small. Since this control removes the finite timing resolution of the time to spike encoder and bypasses the reservoir dynamics, a large improvement would be expected if the encoder resolution itself was making NARMA5 much harder to predict. This is not observed.

Overall, these two tests indicate that, at least for NARMA5 with the present time to spike input encoding, the finite timing resolution of $W_{\mathrm{enc}} = 10$ does not appear to be a major source of prediction error. Increasing the encoding window does not improve the reservoir based readout under the fixed reservoir parameters used here, and replacing the spike input history with the original numerical input history only slightly changes the input only linear baseline.

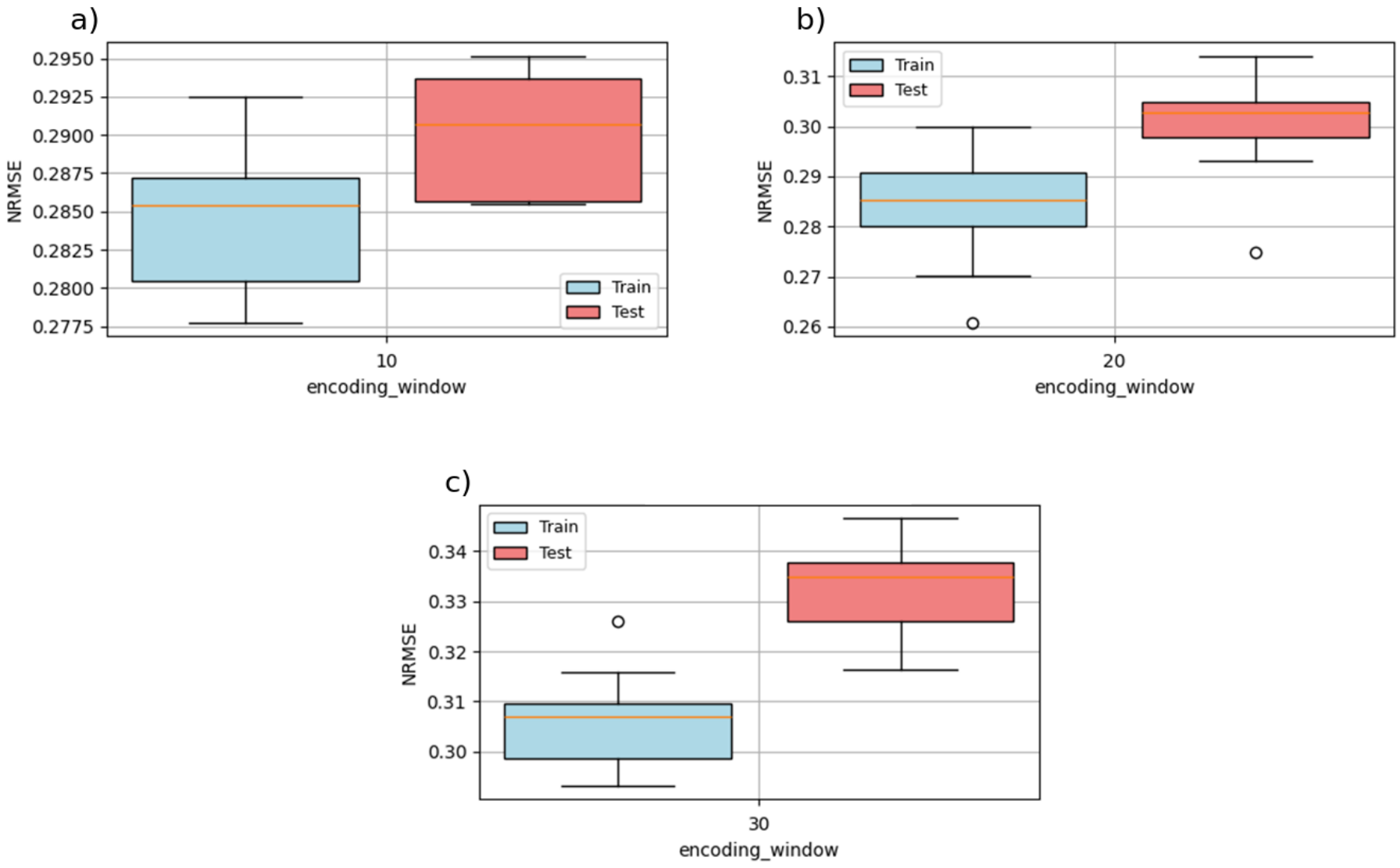

Figure S4: NRMSE on NARMA5 when the time to spike encoding window is increased in the reservoir based sliding window linear readout. a) $W_{\text{enc}} = 10$ with $t_{\text{window}} = 60$. b) $W_{\text{enc}} = 20$ with $t_{\text{window}} = 120$. c) $W_{\text{enc}} = 30$ with $t_{\text{window}} = 180$. The readout window is scaled with the encoding window, so that each setting covers the same number of original NARMA input samples. Increasing W_{enc} does not give a clear reduction in train or test NRMSE.

Additional input encoding experiments

The main experiments use time-to-spike encoding. Since input preprocessing and spike encoding can strongly affect SNN and LSM performance, we repeated the sliding-window linear-readout experiment with two additional encoders: rate encoding and thermometer encoding. These experiments were designed to test whether the benefit of exposing recent reservoir states at the readout is specific to the original time-to-spike encoding.

For the rate encoding experiment, larger scalar input values were represented by higher spike rates within the encoding window. This produces a spike pattern in which the input amplitude is expressed through spike count over a window rather than through the timing of a single spike. For the thermometer encoding experiment, the scalar input value determined how many input neurons received a spike. Larger input values caused spikes to be delivered to more input neurons. We kept the encoding window at $W_{\text{enc}} = 10$ for both additional encoders. For thermometer encoding, this

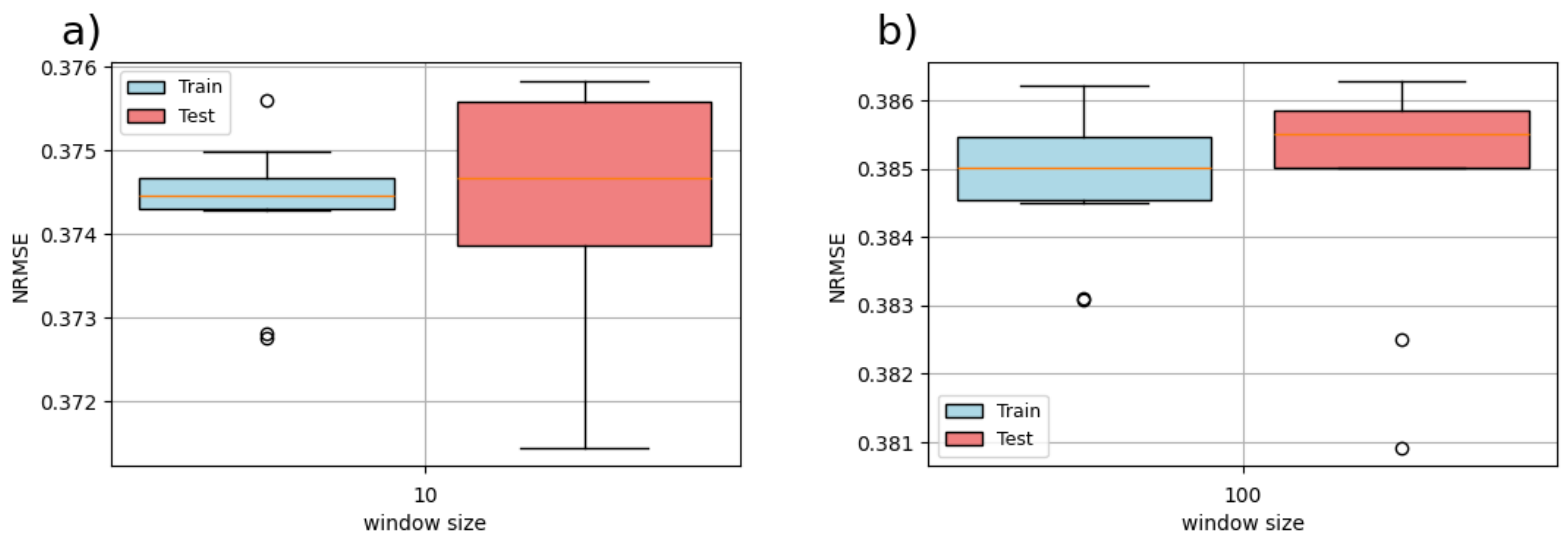

Figure S5: Pure linear regression baseline with and without spike encoding. a) The original numerical NARMA input values are used directly as the readout features. b) The time to spike encoded input history is used. The numerical input window has size 10, while the spike based window has size 100 under $W_{\text{enc}} = 10$, so both settings cover the same number of original NARMA input samples. The two settings give similar NRMSE values.

window does not add temporal coding within a input sample; the same thermometer spike pattern is repeated for ten reservoir simulation steps. Although thermometer encoding does not require multiple time steps to represent the scalar value, we used repetition to stabilise the input drive and keep the temporal scale comparable to the other encoding settings.

Figure S6 shows the rate encoding results. Compared with the time-to-spike setting in the main manuscript, rate encoding gives higher NRMSE across the tested window sizes. The reservoir-based readout is better than the input-history baseline at the shorter windows, however, at $t_{\text{window}} = 100$, the input-history baseline is even slightly better than the reservoir-based readout. Indicating the choice of encoding method could sometime disturb the computation of the reservoir.

Figure S7 shows the thermometer encoding results. Compared with the time-to-spike encoding used in the manuscript, thermometer encoding gives lower NRMSE for the reservoir-based readout and a larger gap to the matched baseline at the largest windows. This extends the proposed method beyond a specific encoder, showing that the proposed sliding-window readout can improve performance across difference encoding schemes.

Time-series comparison of the input-history baseline and reservoir readout

The aggregate NRMSE results in the main text show the overall performance difference between the pure linear regression baseline and the reservoir based readout across random seeds. However, aggregate error alone does not fully show how the predictions behave over time. We therefore provide additional prediction and target traces, together with running NRMSE curves, for two representative context lengths, $t_{\text{window}} = 50$ and $t_{\text{window}} = 100$.

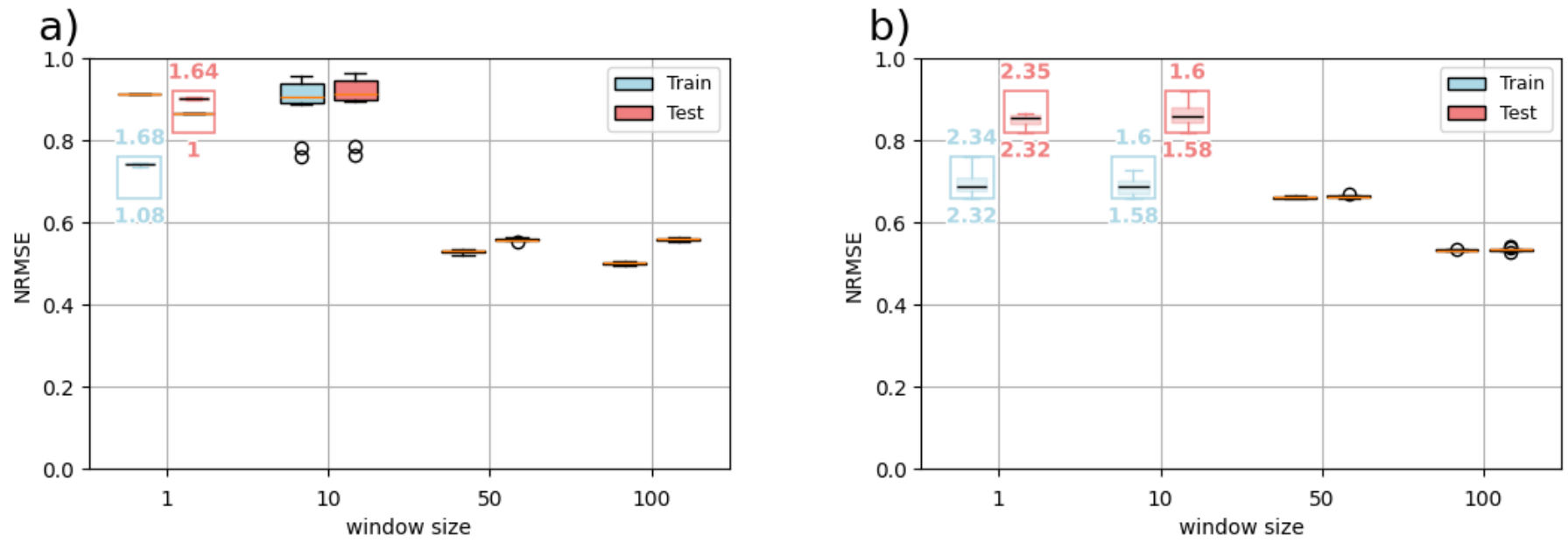

Figure S6: NARMA5 prediction performance under rate encoding. a) Sliding-window linear readout trained on reservoir spike states. b) Pure linear-regression spike baseline that bypasses the reservoir and is trained directly on the rate-encoded input history. Each box summarises 10 random seeds. A mixed y-axis is used: values above the main $[0, 1]$ NRMSE range are plotted in a compressed upper band and annotated with their true values. Rate encoding gives clearly higher error than the time-to-spike setting reported in the main manuscript. Increasing t_{window} still reduces NRMSE at long windows, but the reservoir-based readout does not preserve the clear advantage over the pure input-history baseline; at $t_{\text{window}} = 100$, the pure baseline is comparable to or slightly better than the reservoir readout.

The prediction and target traces show how closely each model output follows the target signal over the displayed test segment. The running NRMSE curves are computed from all prediction and target samples between the start of the displayed segment and the current time step. They provide a complementary summary of the prediction error over time, making it easier to assess whether one method maintains a lower error more consistently across the segment and whether its running error curve stabilises at a lower level.

Figure S8 shows the comparison at $t_{\text{window}} = 50$. The first few points of the running NRMSE are abnormally high because the metric is computed from only a small number of accumulated samples at the beginning of the displayed segment. Since target standard deviation used to normalise the NRMSE can be very small at the beginning and make the metrics big. After this initial region, the reservoir-based readout maintains a lower running NRMSE and shows visibly smaller fluctuations than the input-history baseline. This indicates that its advantage is not just a lower final error, but also a more stable and consistent error across the segment.

Figure S9 shows the larger-window case. At this context length, both methods obtained a smaller NRMSE given they have explicit access to longer history. Even so, the running NRMSE plot shows that the reservoir-based readout maintains a lower running NRMSE across the segment, while the input-history baseline settles at a higher error level and more fluctuate.

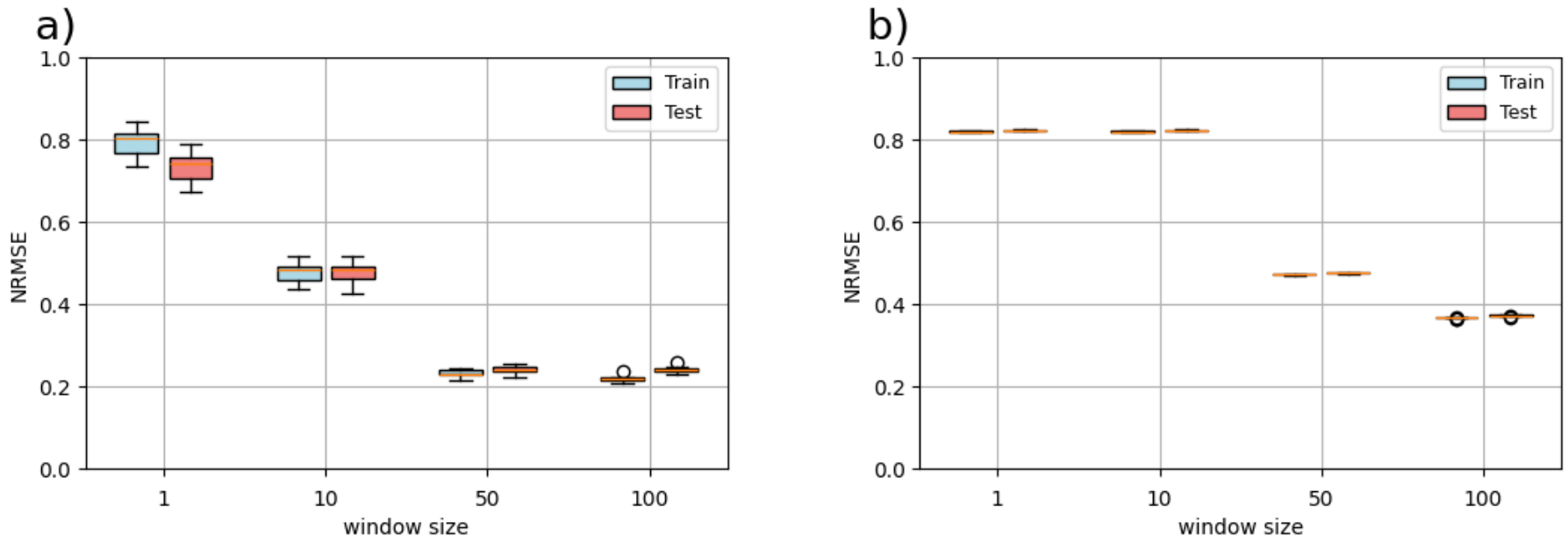

Figure S7: NARMA5 prediction performance under thermometer encoding. a) Sliding-window linear readout trained on reservoir spike states. b) Pure linear-regression spike baseline that bypasses the reservoir and is trained directly on the thermometer-encoded input history. The encoding window was kept at $W_{\mathrm{enc}} = 10$; for thermometer encoding this means that the same population-code input pattern is repeated for ten reservoir simulation steps. Under this encoder, the reservoir-based readout improves strongly with larger windows and achieves substantially lower NRMSE than the matched pure input-history baseline at $t_{\mathrm{window}} = 50$ and 100.

Input-side tapped-delay-line ablation

To test whether the proposed readout-side window can be replaced by a classical input-side tapped delay line, we added a control in which the encoded input spike train was expanded into delayed input channels before entering the reservoir. Each delay step was implemented as a separate input channel with its own randomly initialized input weights to the reservoir. The readout was then trained on the current reservoir state. We also tested a combined condition in which the same input-side delay line was used together with the proposed sliding-window readout.

Figure S10 shows that the input-side tapped delay line does not reproduce the improvement obtained by the proposed readout-side window. As t_{window} increases, the reservoir-state sliding-window readout improves substantially, whereas the input-side delay-line condition degrades toward near-random prediction. The combined condition also does not outperform the readout-side window alone.

Although input delay lines and delay-coordinate ideas have been shown to be effective in classical ESN and reservoir-computing settings [2], this control highlights an important difference between applying a delay line before a continuous-valued ESN and before a spiking LSM. In a standard ESN, the delayed inputs are transformed into continuous reservoir activations that are directly available to the readout. In our LSM setting, the delayed spike channels first pass through fixed random input projections and the LIF threshold-reset reservoir dynamics, while the readout observes only the current binary spike state. Thus, increasing the input-delay window does not simply provide a richer delay vector to the decoder; it changes the reservoir dynamics and can leave useful differences hidden in subthreshold membrane states or mixed into

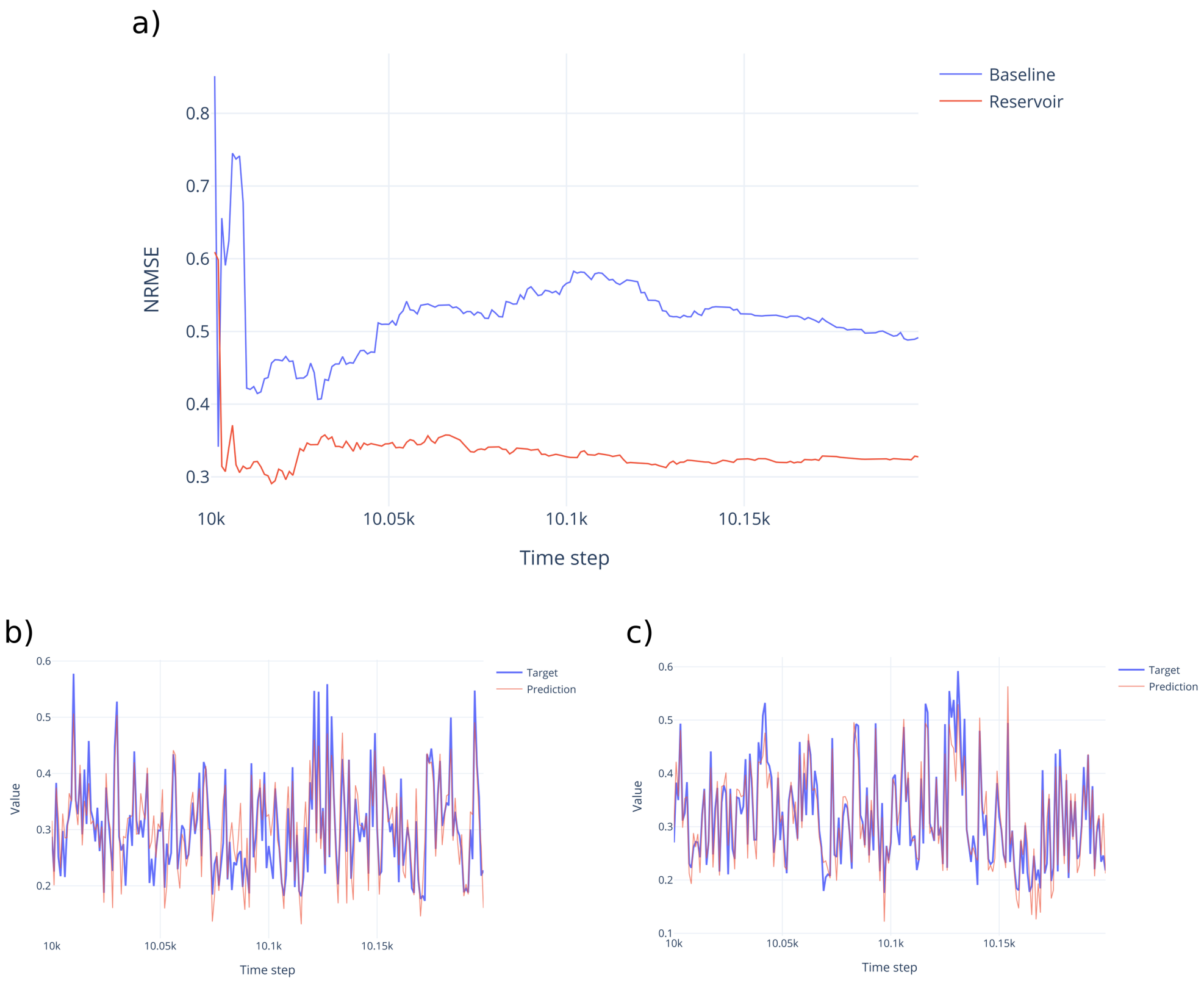

Figure S8: Time-series comparison for $t_{\text{window}} = 50$, corresponding to five NARMA input steps with $W_{\text{enc}} = 10$. a) Running NRMSE over the displayed test segment. b) Prediction–target trace for the input-history baseline. c) Prediction–target trace for the reservoir-based readout. For each method, the displayed run is selected as the best-performing run among ten random seeds for that condition, according to its overall test NRMSE.

spike patterns that are not linearly decodable from the instantaneous state. This helps explain why the input-side tapped delay line degrades as t_{window} increases, rather than reproducing the improvement obtained by the readout-side window. The proposed sliding-window readout avoids this issue by leaving the input stream, input weights, recurrent connectivity, and reservoir dynamics unchanged, and by making the recent reservoir spike trajectory directly available to the decoder as a plug-in readout-side modification.

Reservoir size versus readout temporal context

The main experiments in this work use a reservoir with 400 neurons together with an explicit sliding-window readout. To test whether the observed improvement could

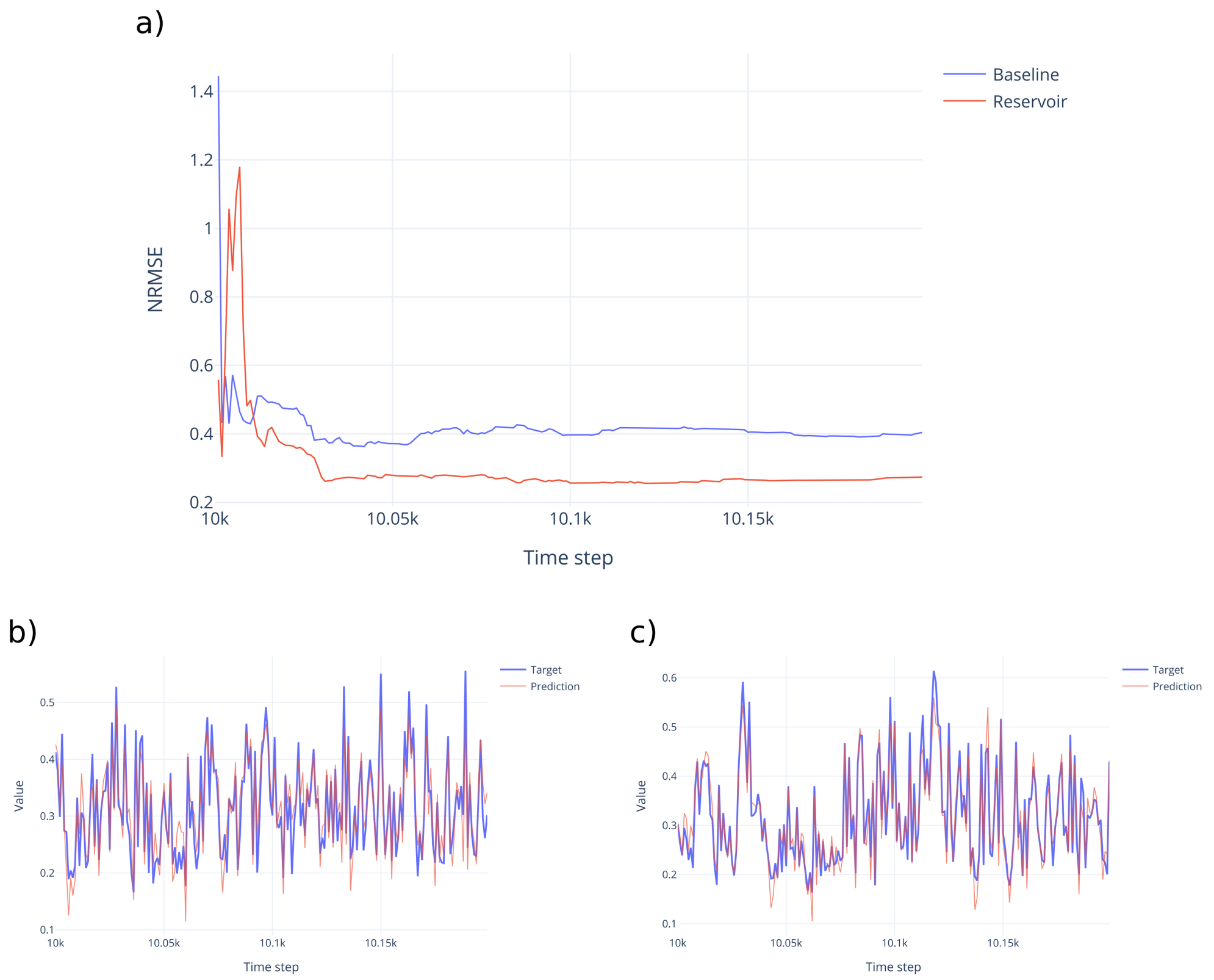

Figure S9: Time-series comparison for $t_{\text{window}} = 100$, representing a larger-context setting beyond the NARMA5 memory scale. a) Running NRMSE over a test segment. b) Prediction–target example for the input-history baseline. c) Prediction–target example for the reservoir-based readout. As in Figure S8, each displayed run is selected as the best-performing run among ten random seeds for the corresponding method and window length.

instead be recovered simply by increasing the size of the recurrent reservoir, we performed an additional comparison between this compact long-window configuration and larger reservoirs decoded from shorter temporal contexts.

This comparison evaluates the relationship between reservoir size and explicit temporal context at the readout. We compare the compact configuration used in the main experiments, a 400 neurons reservoir with $t_{\text{window}} = 50$, with larger reservoirs decoded from shorter temporal contexts. The larger reference configurations include a 900 neurons reservoir with $t_{\text{window}} = 20$ and a 2500 neurons reservoir with the standard stepwise readout, $t_{\text{window}} = 1$. This provides a cost-performance comparison between recurrent reservoir scaling and readout-side temporal context.

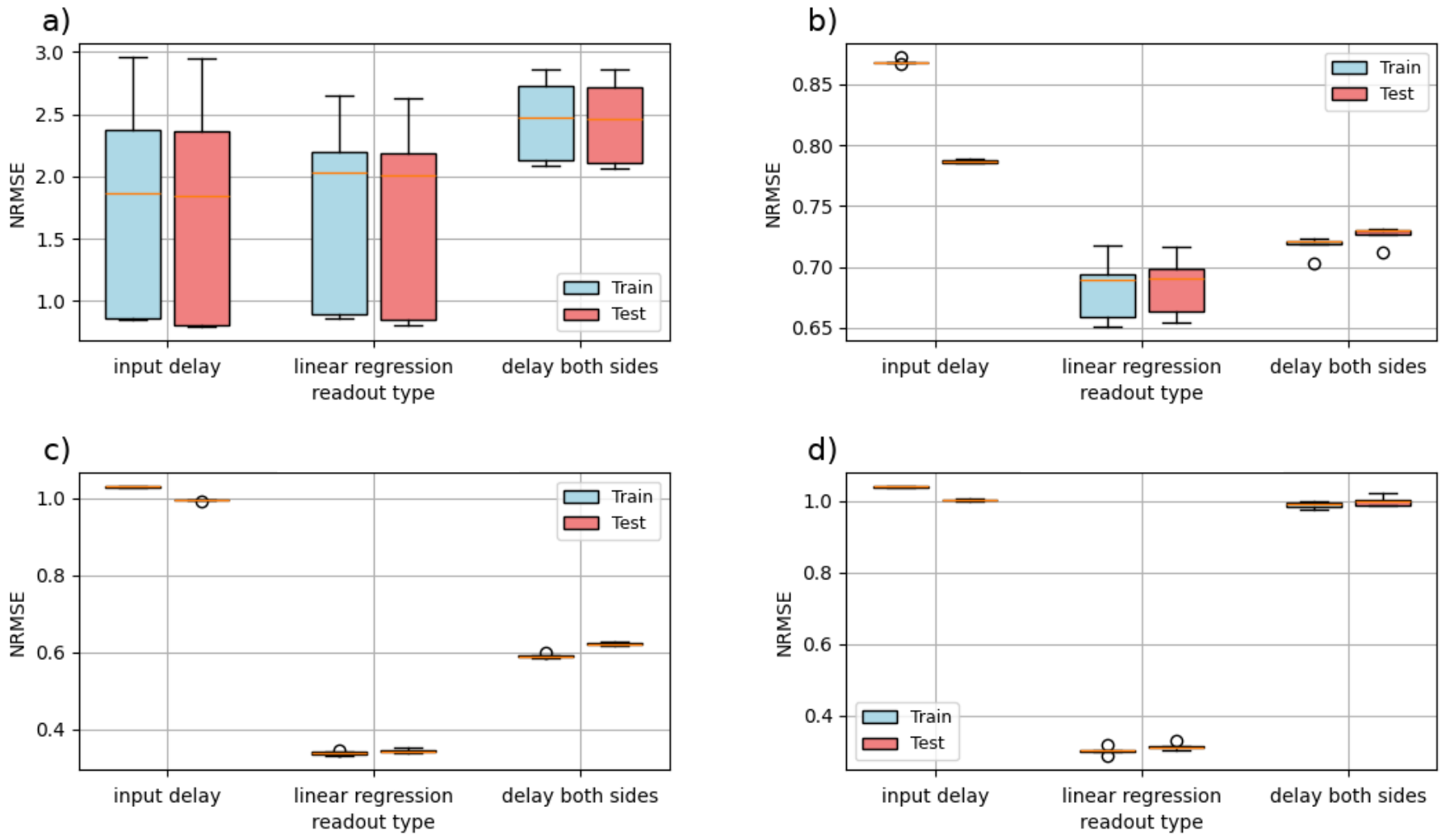

Figure S10: Input-side tapped-delay-line control on NARMA5. a) $t_{\text{window}} = 1$. b) $t_{\text{window}} = 10$. c) $t_{\text{window}} = 50$. d) $t_{\text{window}} = 100$. We compare an input-side tapped delay line (Input TDL), the proposed reservoir-state sliding-window readout (Reservoir-state SW readout), and a combined condition using both input-side delay and the readout-side sliding window (Input TDL + SW readout). The context length t_{window} is measured in reservoir simulation steps.

For each reservoir size, the distance scale parameter s in the spatial connectivity rule was selected from an independent distance scale sweep. The values used here therefore correspond to the best tested connectivity density for each reservoir size.

Figure S11 compares the compact long-window reservoir used in the main experiments with larger reservoirs decoded from shorter temporal contexts. The compact configuration contains 400 recurrent neurons and uses $t_{\text{window}} = 50$. It achieves the lowest NRMSE among the tested configurations. By comparison, the 900-neuron reservoir with $t_{\text{window}} = 20$ gives higher error, and the 2500-neuron stepwise reservoir gives the highest error.

This result shows that, for the NARMA5 benchmark, increasing the recurrent reservoir size under a shorter readout window is less effective than providing a longer temporal context to the readout.

These results also provide a structural cost-performance comparison between recurrent reservoir scaling and readout-side temporal context. The comparison supports

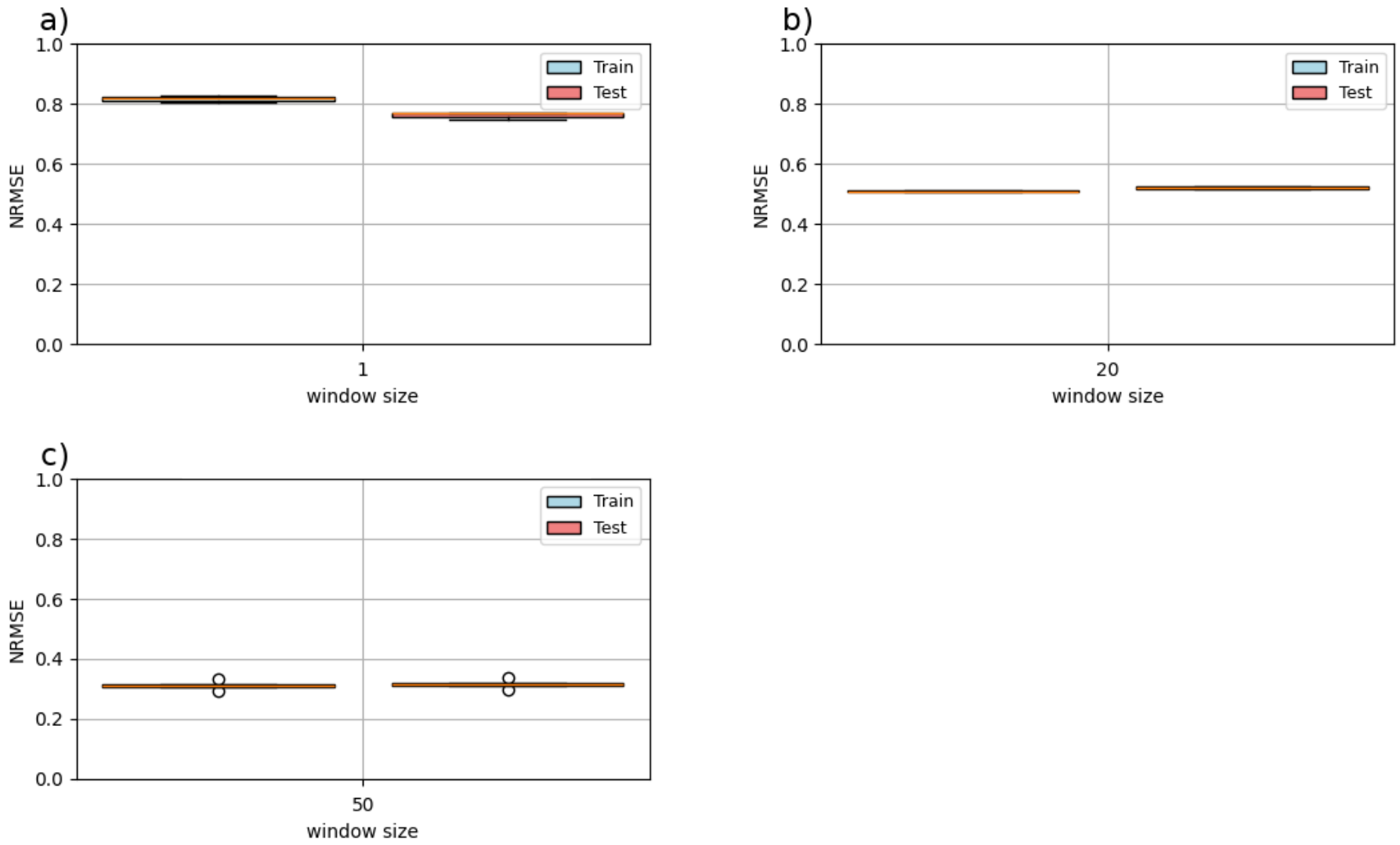

Figure S11: NARMA5 prediction performance for three reservoir size and readout context configurations. a) 2500 recurrent neurons with the standard stepwise readout, $t_{\text{window}} = 1$. b) 900 recurrent neurons with $t_{\text{window}} = 20$. c) The compact configuration used in the main experiments, containing 400 recurrent neurons with $t_{\text{window}} = 50$. For each reservoir size, the distance scale is selected from an independent distance scale sweep. Each box summarizes 10 random seeds.

the main design choice of using a compact spiking reservoir together with an explicit sliding-window readout for NARMA5 prediction.

References

[1] G. Holzmann, "Echo state networks in audio processing," 2007.

[2] A. G. Hart, J. L. Hook, and J. H. P. Dawes, "Embedding and approximation theorems for echo state networks," *Neural Networks*, vol. 128, pp. 234–247, 2020.
